# miR29b regulates aberrant methylation in *In-Vitro* diabetic nephropathy model of renal proximal tubular cells

**DOI:** 10.1371/journal.pone.0208044

**Published:** 2018-11-29

**Authors:** Piyush Gondaliya, Aishwarya Dasare, Akshay Srivastava, Kiran Kalia

**Affiliations:** 1 Department of Biotechnology, National Institute of Pharmaceutical Education and Research- Ahmedabad; 2 Department of Medical Devices, National Institute of Pharmaceutical Education and Research- Ahmedabad; University of Louisville, UNITED STATES

## Abstract

The role of DNA methylation has not been enough explored in pathophysiology of diabetic nephropathy (DN). However, according to recent reports it has been inferred that hypermethylation could be one of the principle cause associated with the enhancement of DN. An interrelationship between miR29b and DNA methylation has been studied via *in-silico* analysis. We have validated that miR29b prominently targets DNA methyl transferase (DNMT), specifically DNMT1, DNMT3A and DNMT3B. We have developed *in vitro* DN model using renal proximal tubule epithelial cells (RPTECs), contributed to a significant alleviation in RNA and protein expression levels of DNMT3A, DNMT3B and DNMT1. The developed model has also demonstrated downregulation in expression of miR29b. Our studies have also suggested that miR29b targets DNMT1 via targeting its transcription factor SP1. In addition to this, downregulation of a specific biomarker for kidney injury, tubular kidney injury molecule-1 (KIM-1) and fibrosis causing glycoprotein i.e. fibronectin, was also demonstrated. Hence, the developed model revealed that hypermethylation is a key factor incorporated in DN, and miR29b could effectively ameliorate defensive actions in DN pathogenesis.

## Introduction

Diabetes mellitus is a rapidly growing concern around the globe. It is associated with several microvascular and macrovascular complications. Diabetic nephropathy (DN) is one of the major microvascular complication of diabetes which is a major cause behind development of end-stage renal disorder (ESRD). The disease ranges from microalbunumeria to progressive chronic kidney disease [1, 2]. The pathogenesis of diabetic nephropathy includes a complex interaction between metabolic and haemodynamic factors that leads to an increase in formation of advanced glycation end products (AGE) as well as increase in factors promoting growth like angiotensin-II (Ang-II) and transforming growth factor beta1 (TGF-β1) [3, 4]. Collective evidences have suggested that the factors associated with DN pathogenesis are influenced by various epigenetic modifications such as methylation on the promotor region of DNA, non-coding RNAs and histone modulations. These factors have showed their pivotal role in DN pathogenesis by a second layer of gene regulation, activation of various gene signaling pathways and ultimately causing vascular complications [5, 6]. Among various epigenetic modifications, abberant DNA methylation contributes to increased prevalance of type I and type II diabetes and is found to be a major regulator of transcriptional activity in DN [7–10]. Genome wide association studies (GWAS) suggest that hypermethylation of genes such as *NPHP4*, *IQSEC1* and *TCF3* [11] are involved in pathways that trigger epithelial to mesenchymal transitions and kidney fibrosis while methylation of *NRBF2; RUNX3; ZBTB5; ZNF639* are known to regulate transcriptional activity [9].

microRNAs (miRNAs) are endogenously produced small, non-coding RNAs of 21–25 nucleotide length, affecting every cellular function by modulating gene expressions. Amongst them, miR29 family is the most excessively expressed miRNA in liver, kidney and pancreas in humans and mice affecting glucose and lipid metabolism and insulin irresponsiveness [12, 13]. In diabetic nephropathy, miR29b is remarkably downregulated in response to AGE and TGF-β1-Smad3 signalling which promotes renal fibrosis. Downregulation of miR29b was found to be associated with higher expression of collagen type1 (COL-I) and collagen type 4 A1(COL4A1) in extracellular matrix (ECM) [14–16]. After going through the repertoire of literature, it has been observed that miR29b regulates abnormal methylation induced in various pathogenic diseased conditions such as lung cancer [17], acute myeloid leukaemia [18] as well as hepatocellular cancer [19].

Amongst DNA methyl transferases, DNMT3A, DNMT3B and DNMT1 are the biomarkers specific to methylation, which were shown as one of the contributing factors in developing DN pathogenesis. Different miRNAs showed distinct expression patterns in DN. It has been reported that TGF-β1, a pro-fibrotic cytokine downregulates miR29b expressions in RPTECs conferring to progression in DN. However, the relationship between miR29b downregulation and hypermethylation in DN conditions is yet to be established. With this aim, we hypothesized that there is a correlation between downregulation of miR29b and hypermethylation in DN pathogenesis. Initially, we generated an *in vitro* DN model using RPTECs using combined treatment of DN promoting factors i.e. AGE, high glucose (HG) and Ang-II. Participation of miR29b in aberrant methylation patterns occurring in DN was also investigated in developed *in vitro* DN model. Collectively, our studies have suggested that miR29b might be involved in downregulation of methylation-specific genes (DNMT3A, DNMT3B and DNMT1) as their aberrant expressions contribute to hypermethylation in *in vitro* DN model and thereby their downregulation via miR29b can alleviate the DN conditions.

## Results

### miR29b target prediction by bioinformatics algorithm

**Fig 1:**
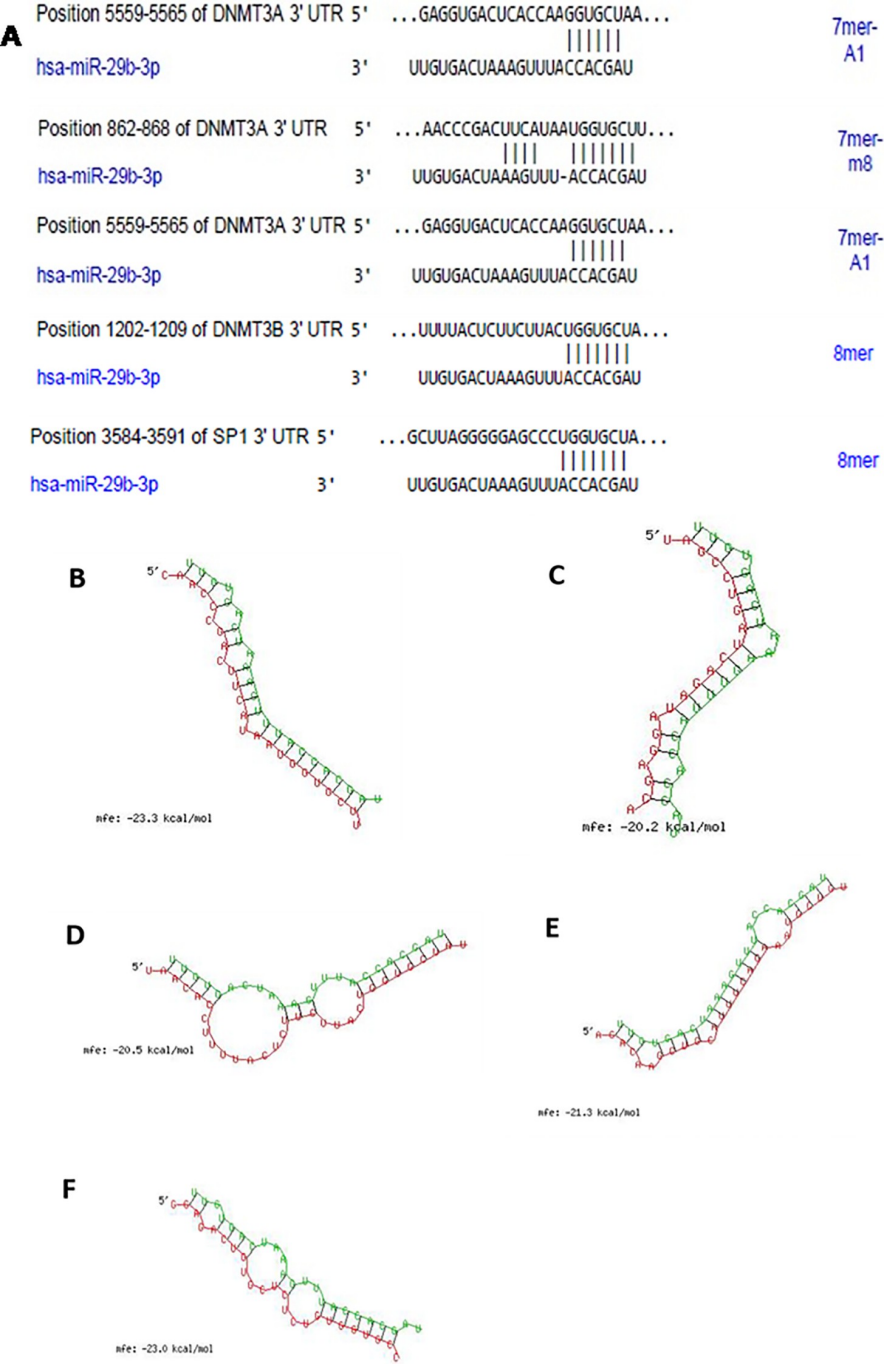
Bioinformatics analysis of miR29b prediction via TargetScan and RNAhybrid tool. **A:** Binding position prediction of 3’UTRs of DNMT3A, DNMT3B and SP1 with miR29b by TargetScan. **B and C:** Secondary structure prediction of DNMT3A mRNA with miR29b by RNAhybrid. **D and E:** Secondary structure prediction of DNMT3B mRNA with miR29b by RNAhybrid. **F:** Secondary structure prediction of SP1 mRNA with miR29b by RNAhybrid.

Three different target sites in 3’UTRs of DNMT3A and one target site for DNMT3B and Specific Protein (SP1), a transcription factor for DNMT1 were predicted based on TargetScan (www.targetscan.org/) analysis (Fig 1A). The secondary structures of miRNA with mRNA of DNMT3A (Fig 1B and Fig 1C), DNMT3B (Fig 1D and Fig 1E) and SP1 (Fig 1F) were obtained from RNAhybrid tool. The sequences shown in red colour are the miR29b sequences complementary to the 3’UTRs of DNMTs shown in green colour. All the targets have demonstrated minimum binding energy of -20 kcal/mol, indicating a stable complex formation between miR29b and 3’UTRs of DNMTs.

### Evaluation of miR29b role in high glucose, AGE and Ang II induce diabetic nephropathy in renal proximal tubule cells

**Fig 2:**
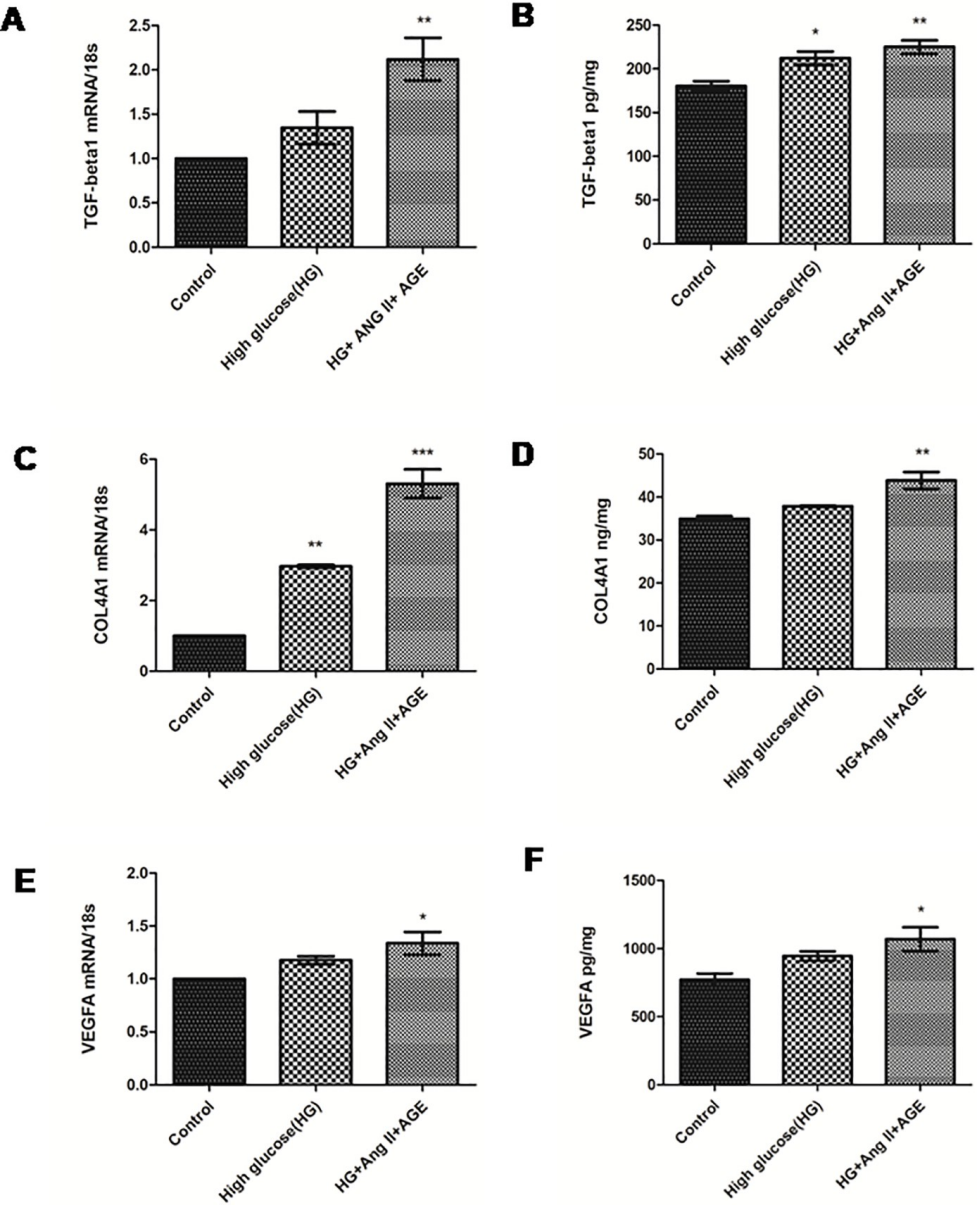
Effect of high glucose, angiotensin II and AGE on RPTECs. **A and B:** Assessment of mRNA expression profiles and protein levels of TGF-β1 in control, HG and cells treated with HG (30 mM), Ang II (1 μM) and AGE products (150 μg/ml) respectively. **C and D**: Assessment of mRNA expression profiles and protein levels of VEGF-A in control and cells treated with HG (30 mM), Ang II (1 μM) and AGE product (150 μg/ml) respectively and **E:** Assessment of mRNA level and protein level of Col4A1 in control and cells treated with HG (30 mM), Ang II (1 μM) and AGE product (150 μg/ml) respectively. Results are represented as mean ± S.D. *(n = 3)*, *p<0.05, **p < 0.01, ***p < 0.001 which shows significant difference in comparison with control group.

Thee different concentrations (0.1, 0.5 and 1 μM.) of Ang-II and AGE (50, 100 and 150 μg/ml) were taken to check the expression of TGF-β1, vascular endothelial growth factor-A (VEGF-A) and COL4A1 for the development of *in vitro* DN model. Based on the mRNA expression profile using RT-PCR, we have finalized 1 μM Ang-II concentration and 150 μg/ml AGE concentration for further standardization. Later, RPTECs were divided in three different combinations to standardize the *in vitro* model by checking expression of TGF-β1 and COL4A1. Real time polymerase chain reaction (RT-PCR), analysis of RPTECs induced by all the three components have demonstrated higher expression of TGF-β1 and COL4A1 (S1 Fig). RPTECs were cultured in three different combinations of stress; 1) HG and Ang-II, 2) HG and AGE, 3) HG, Ang-II and AGE, and these three combinations were compared with high glucose stress model *in vitro*. RT-PCR studies of TGF-β1, VEGF-A and COLA1 verified their expression profiles involved in DN pathogenesis *in vitro* (Fig 2). Hence, we found that supplementing HG (30 mM) along with Ang-II (1 μM) (Sigma) and AGE products (150 μg/ml) (Sigma) at 37°C for 48 h with 5% CO_2_ could provide the appropriate conditions to generate a DN model.

### Expression profiles of oxidative stress markers and miR29b and their targets in diabetic nephropathy model

**Fig 3:**
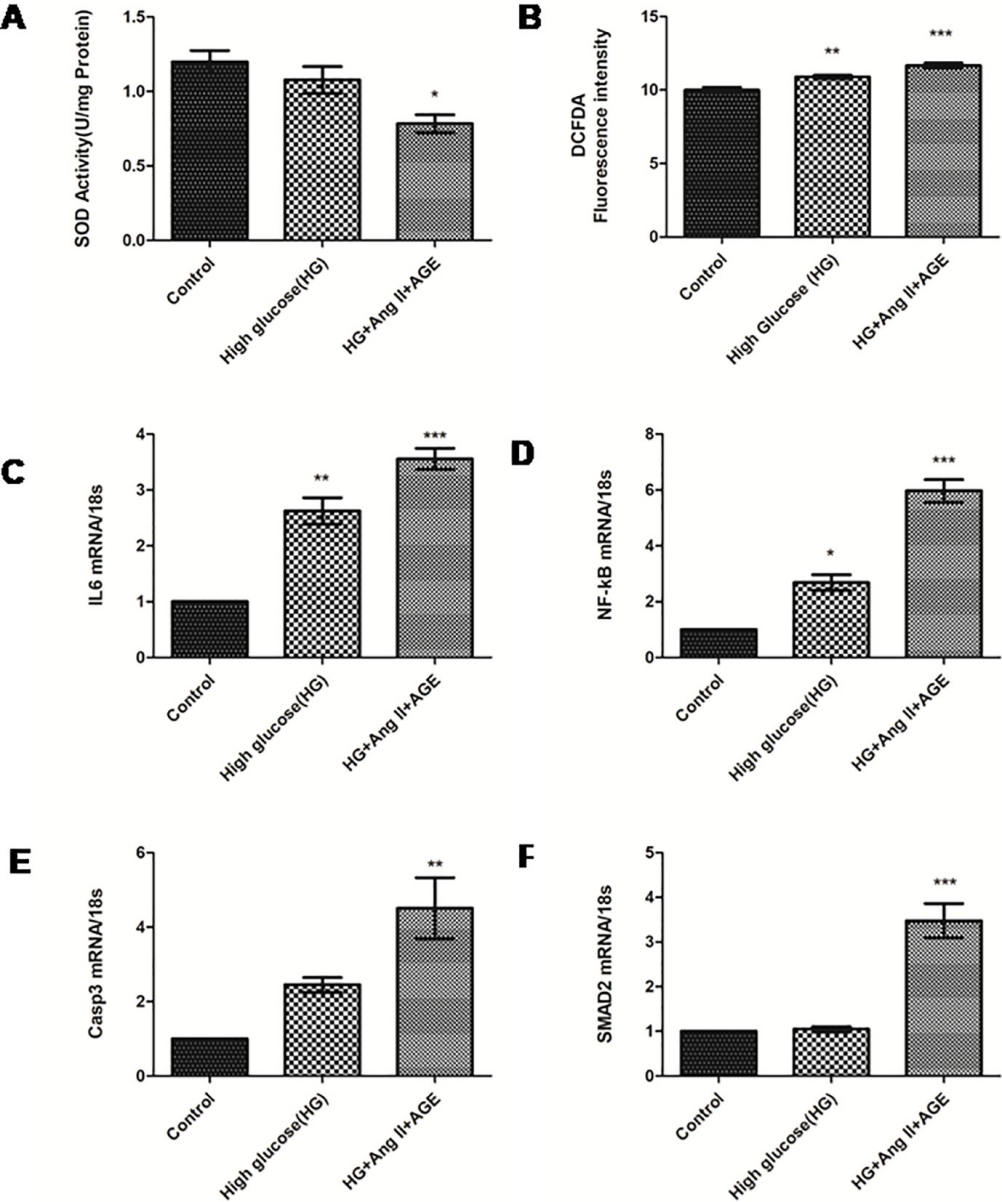
Measurement of oxidative stress and expression profile of their markers. **A and B**: Measurement of SOD activity and intracellular ROS production by DCFDA dye in control, HG and cells treated with HG, Ang II and AGE product respectively **C, D, E, F:** Assessment of mRNA level of IL6, NF-κβ, Caspase3 and SMAD2 in control, high glucose and cells treated with HG, Ang II and AGE product respectively. Results are represented as mean ± S.D. *(n = 3)*, *p<0.05, **p < 0.01, ***p < 0.001 which shows significant data when compared with control group).

In this study, rise in the production of reactive oxygen species (ROS) was found in RPTECs treated with HG, AGE and Ang-II as compared to untreated RPTECs (Fig 3A). A gradual decrease in superoxide dismutase (SOD) activity also indicates an increase in ROS production (Fig 3A and Fig 3B). This subsequently contributed to an increase in inflammatory cytokines like interleukin-6 (IL-6) which is analysed by qRT-PCR (Fig 3C). The role of nuclear factor kappa β (NF-κβ) and caspase 3 (CASP3) were also quantified in RPTECs based *in vitro* DN model. It has revealed enhanced level of NF- κβ in comparison with only high glucose induced model (Fig 3D and Fig 3E). Expression of Smad2 was also increased in RPTECs treated with HG, AGE product and Ang-II compared to only HG model (Fig 3F).

### Advanced glycation end products, high glucose and angiotensin-II induce hypermethylation via downregulation of miR29b

**Fig 4:**
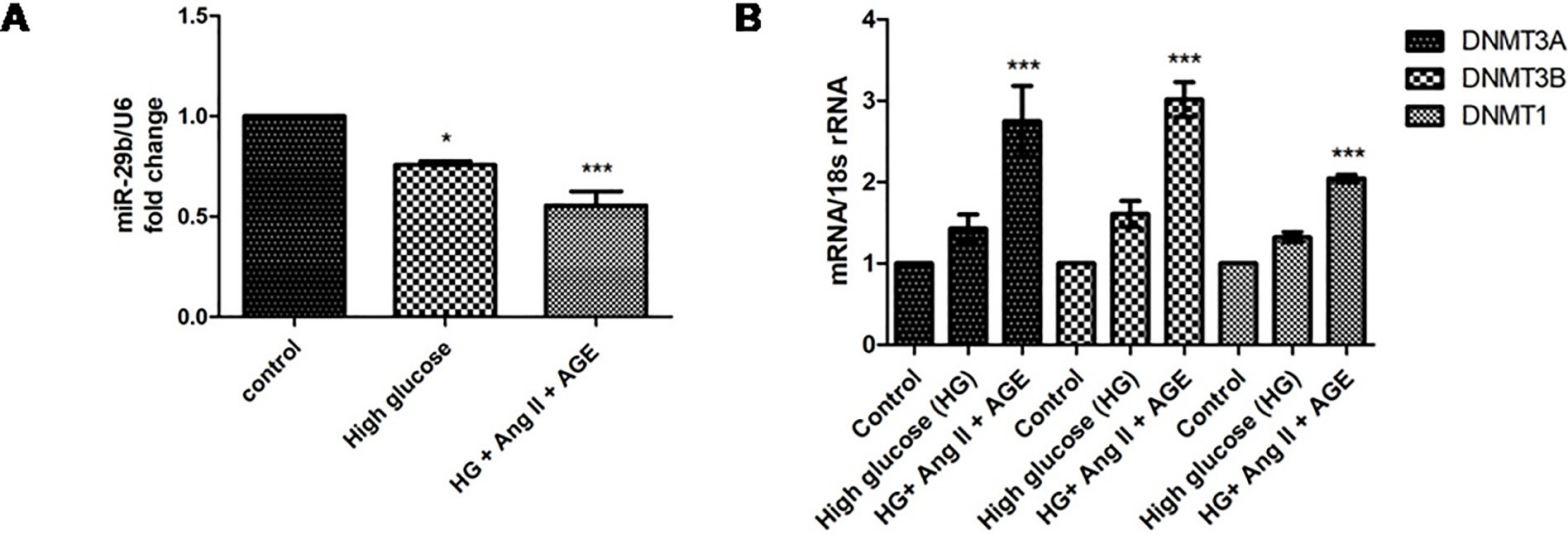
Effect of high glucose, angiotensin II and AGE product on expression levels of miR-29b and their targets. **A**. Downregulation of miR29b level in RPTECs in diabetic nephropathy model. **B.** Quantification of mRNA levels of DNMT3A, DNMT3B and DNMT1 in Diabetic Nephropathy model. Results are represented as mean ± S.D. *(n = 3)*, *p<0.05, **p < 0.01, ***p < 0.001 which shows significant difference when compared with control group).

The developed *in vitro* DN model have been investigated to demonstrate the mRNA expression of miR29b and establish a relationship between expression of miR29b and hypermethylation. In our results, significant downregulation of miR29b was observed in *in vitro* DN model compared to HG treated model (Fig 4A). It was also observed that expression levels of DNMT3A, DNMT3B and DNMT1 were significantly elevated in model generated with HG, AGE and Ang-II (Fig 4B).

### The expression profiles of DNMT3A, DNMT3B and SP1 regulated by miR29b in diabetic nephropathy

**Fig 5:**
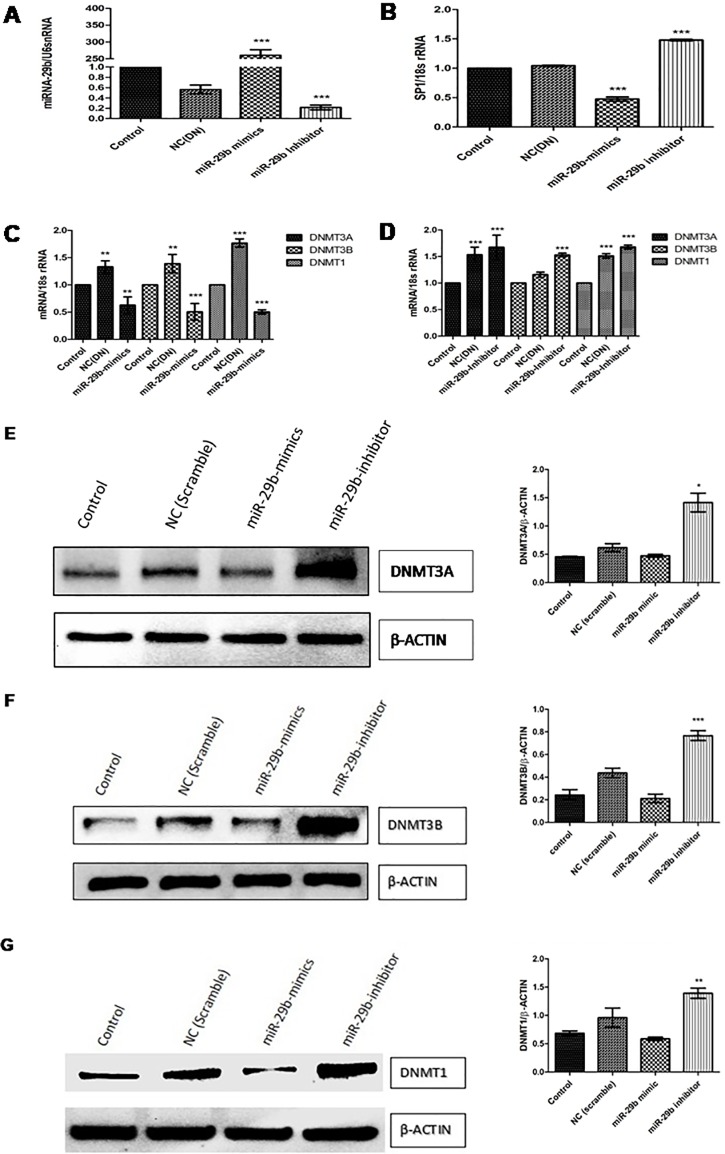
miR-29b modulates expression of DNMT3A, DNMT3B,DNMT1 and SP1 in RPTECs. **A:** Transfection efficiency of miR-29b mimics and inhibitor analysed by RT-PCR **B, C and D**: Effect of miR29b mimics and inhibitors on mRNA expression levels of SP1, DNMT3A, DNMT3B and DNMT1 respectively in DN model analysed by Real-time PCR. **E, F and G:** Assessment of protein levels of DNMT3A, DNMT3B and DNMT1 in control, negative control (Transfected with scramble vector) and DN model (transfected with miR29b mimics and inhibitors) performed by Western Blotting (n: 3), * indicates ‘p’ value <0.05, ** indicates ‘p’ value < 0.01, *** indicate p value <0.001.

**Fig 6:**
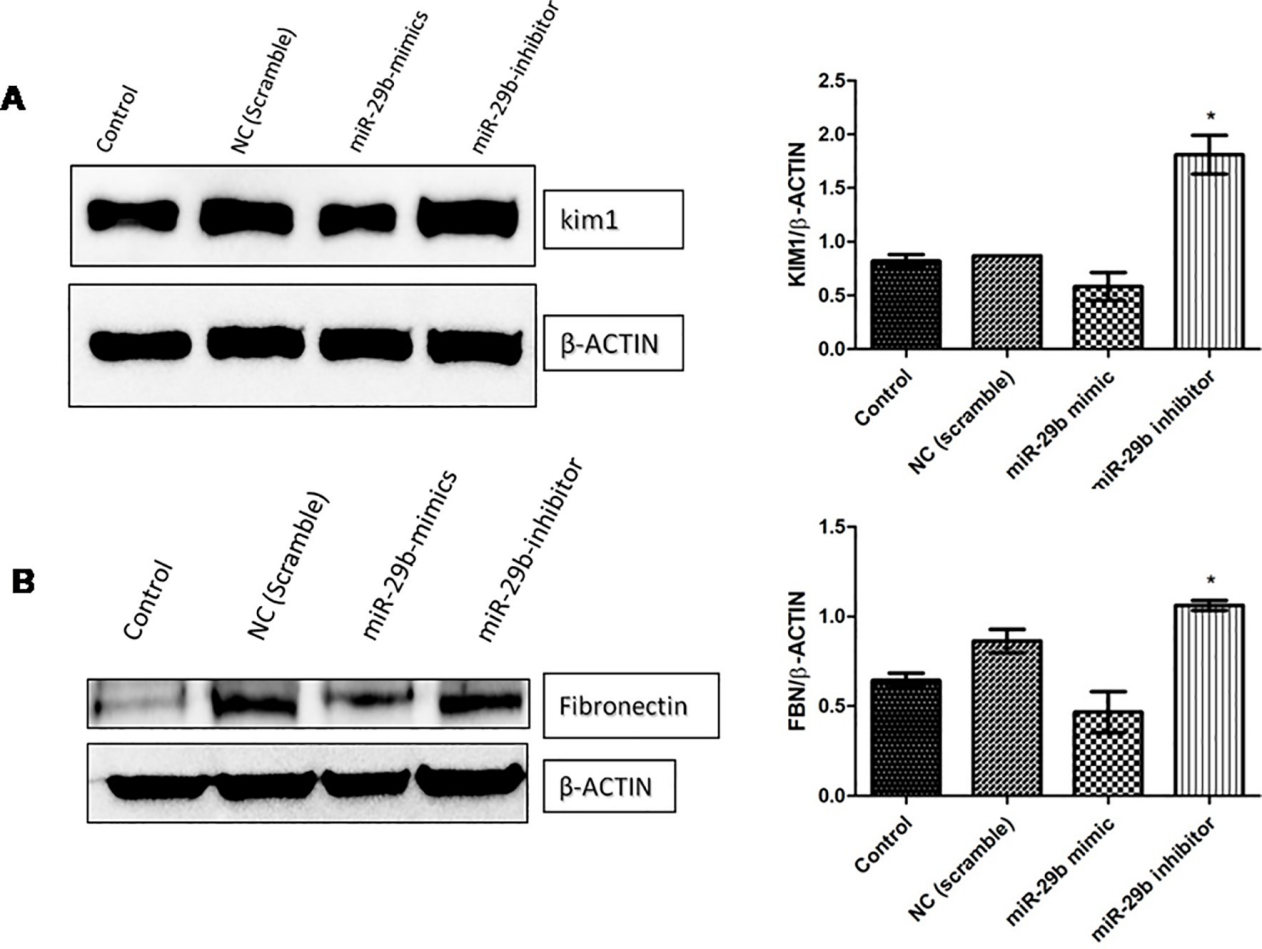
Effect of miR29b mimics and inhibitors on expression profiles of DNMT1 in DN model. **A and B:**Immunofluorescence microscopy as well as quantitative analysis shows that transfection with miR29b mimics attenuated DNMT1 expression level while transfection with miR29b inhibitors showed elevated expression levels of DNMT1. Scale bar, 20 μm. Representative (n = 5), * indicates ‘p’ value <0.05, ** indicates ‘p’ value < 0.01, *** indicate p value <0.001.

**Fig 7:**
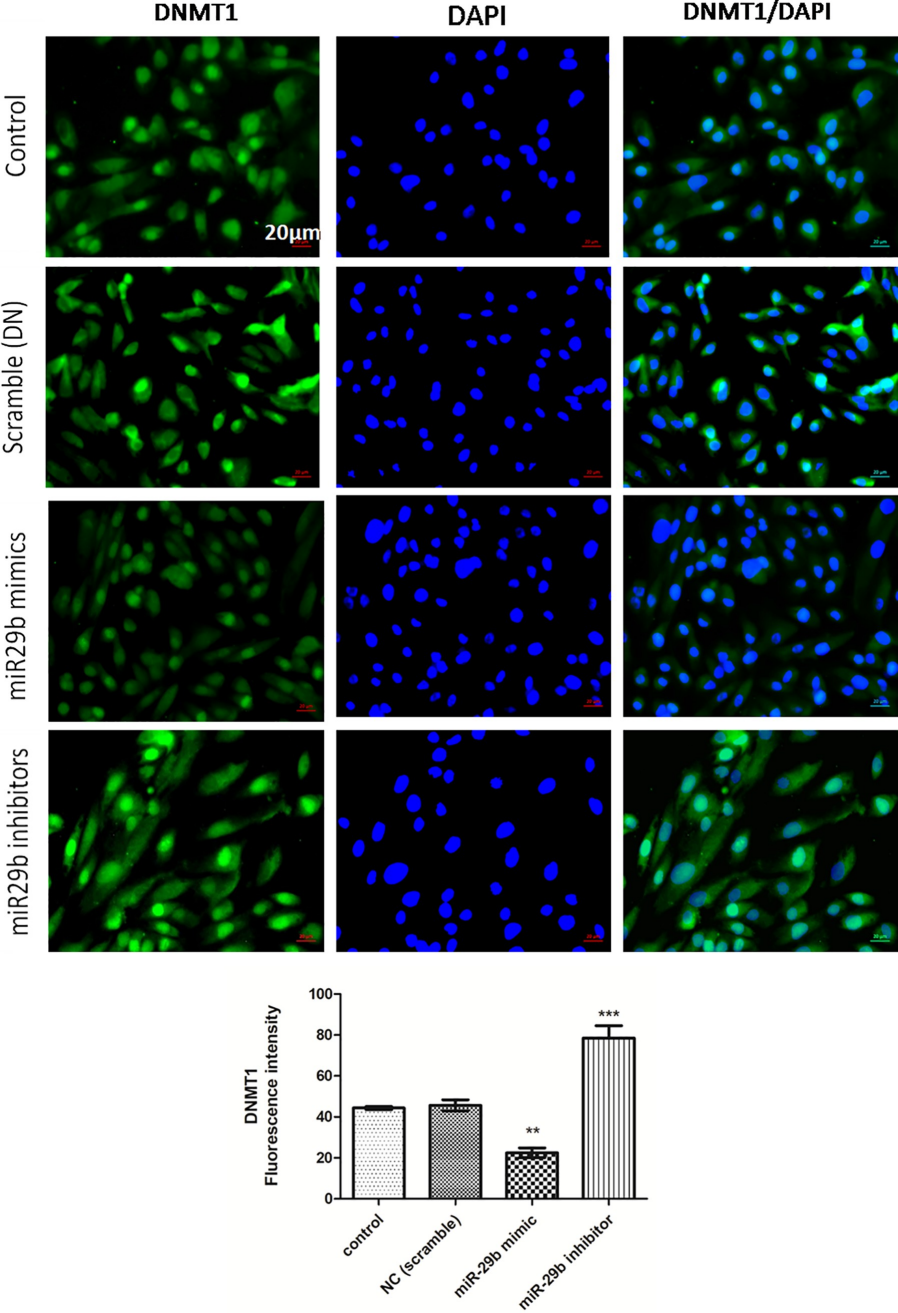
Effect of miR29b mimics and inhibitors on expression profiles of DNMT3B in DN model. **A and B:**Immunofluorescence microscopy as well as quantitative analysis shows that transfection with miR29b mimics attenuated DNMT3B expression level while transfection with miR29b inhibitors showed elevated expression levels of DNMT3B. Scale bar, 20 μm. Representative (n = 5), * indicates ‘p’ value <0.05, ** indicates ‘p’ value < 0.01, *** indicate p value <0.001.

**Fig 8:**
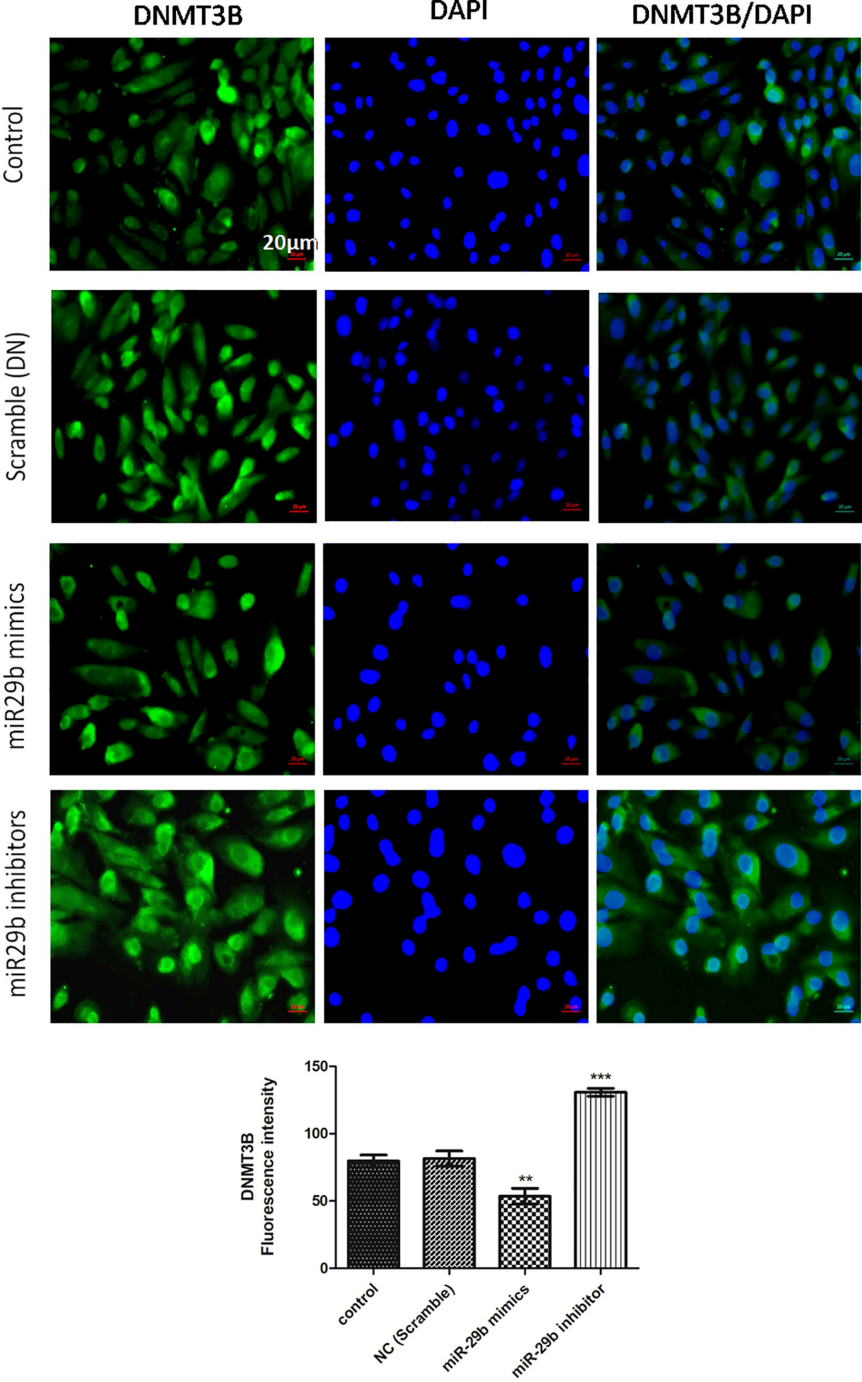
Effect of miR-29b mimics and inhibitor on expression profile of DNMT3A in DN model. **A and B:** Immunofluorescence microscopy as well as quantitative analysis shows that transfection with miR29b mimics attenuated DNMT3A expression level while transfection with miR29b inhibitors showed elevated expression levels of DNMT3A. Scale bar, 18μm. Representative (n = 5), * indicates ‘p’ value <0.05, ** indicates ‘p’ value < 0.01, *** indicate p value <0.001.

**Fig 9:**
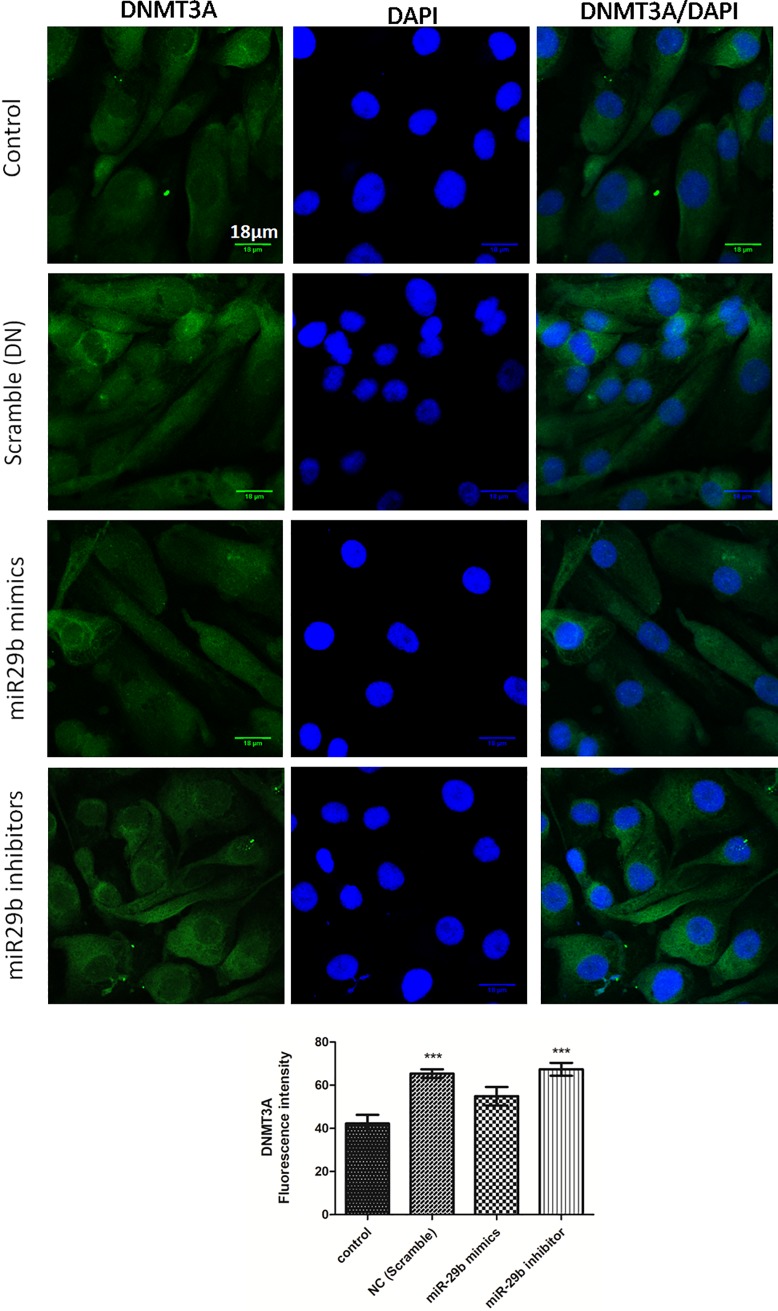
miR-29b decreases fibrosis and tubular injury via downregulation of fibronectin and KIM-1. **A and B:** Effect of miR29b mimics and inhibitors on expression profiles of KIM-1 and Fibronectin in DN model (n = 2), * indicates p value <0.05.

Expression of DNMTs in RPTECs of *in vitro* DN model were checked by transfection of miR29b mimics, miR29b inhibitors and compared with the transfection of scramble miRNA as negative control. The qRT-PCR analysis have showed that miR29b mimics induced downregulation of DNMT3A, DNMT3B and DNMT1 along with the downregulation of SP1 which is a transcription factor required for DNMT1 expression (Fig 5A). On the other hand, miR29b inhibitors induced upregulation of DNMT3A, DNMT3B, DNMT1 and SP1 (Fig 5B–5G). Imunocytochemistry (ICC) of miR29b mimics and inhibitor treated *in vitro* DN model have demonstrated alleviated expression levels of DNMT1 (Fig 6A), DNMT3B (Fig 7A) and DNMT3A (Fig 8A) upon transfection with miR29b mimics while elevation in the expression levels of the same upon transfection with miR29b inhibitors. The fluorescent intensity quantification have shown that upon transfection with miR29b mimics, the expression of DNMT3A, DNMT3B and DNMT1 were attenuated while upon transfection with miR29b inhibitors, expression levels of these markers were alleviated (Figs 7–9).

### miR29b suppresses tubular injury in high glucose, AGE products and angiotensin-II induced diabetic nephropathy in RPTECs

To further examine miR29b role in fibrosis as well as in tubular injury, miR29b mimics and inhibitors was administered in *in vitro* DN model developed using RPTECs. Western blot of fibronectin (FBN) and KIM-1 protein was analyzed which revealed that miR29b mimics alleviated expressions of FBN and KIM-1 whereas miR29b inhibitor increased the expressions of FBN and KIM-1 in DN model (Fig 9A and 9B).

### miR29b directly targets DNMT3A/3B and indirectly targets DNMT1 via SP1

**Fig 10:**
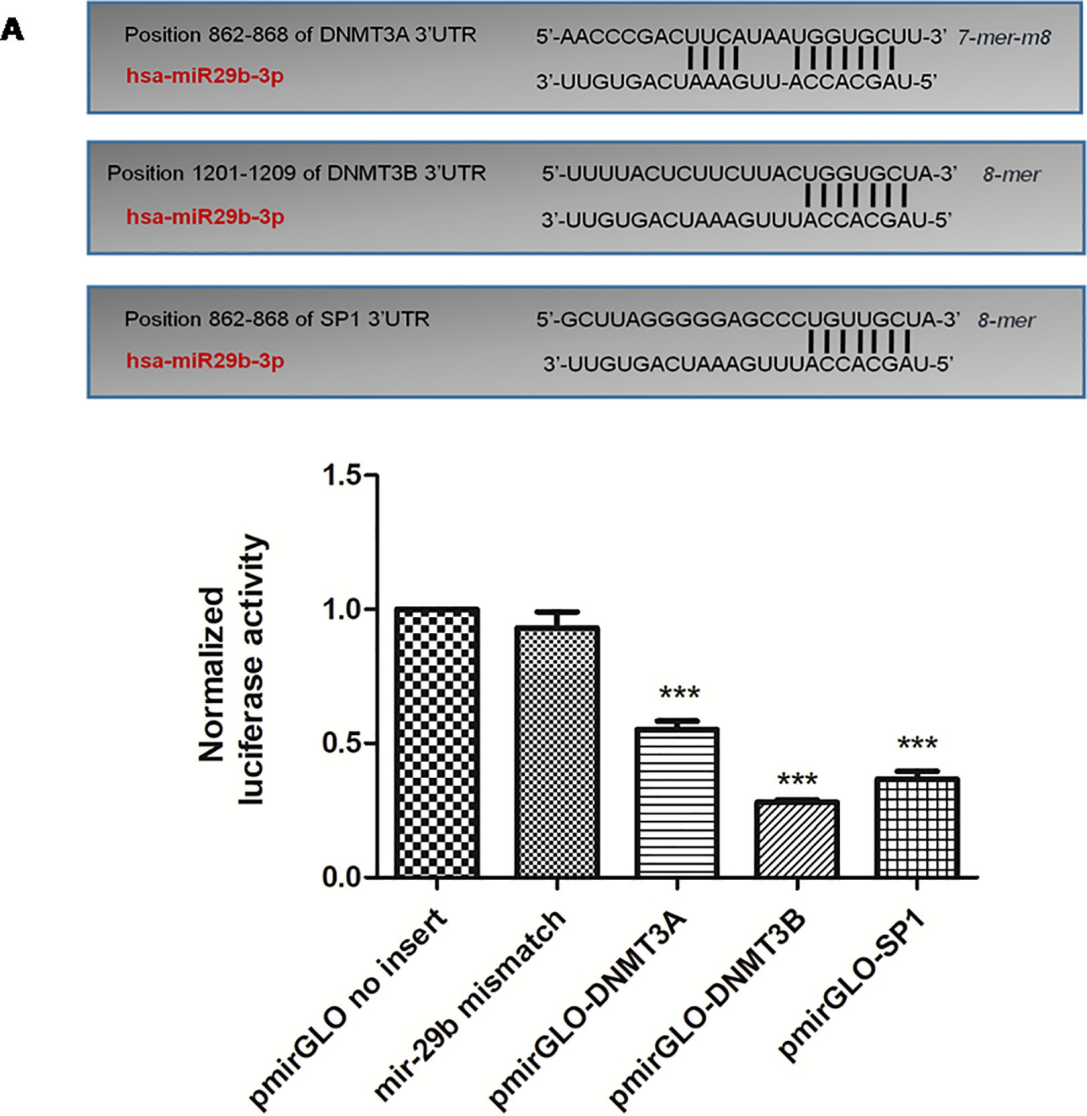
Luciferase activity measurement: The 3’-UTRs of DNMT3A, DNMT3B and SP1 are targets of miR-29b. This figure describes a schematic of binding sites of miR29b with 3’-UTRs of DNMT3A, DNMT3B and SP1. Below is the graphical representation of Dual Luciferase Assay illustrated normalised Luciferase activity of pmirGLO vector (without 3’-UTR insert) and cells transfected with miR29b mismatch sequence (scrambled oligonucleotide). Further, pmirGLO vector with 3 different inserts of DNMT3A, DNMT3B and SP1 were allowed to interact with miR29b mimics independently and their Luciferase activity was examined as shown in this bar graph indicating significant difference in comparison with pmirGLO vector (without insert) and cells transfected with miR29b mismatch sequence (***p< 0.001).

Through *in-silico* analysis and *in vitro* analysis, it was found that DNMT3A, DNMT3B and DNMT1 are the potential targets of miR29b. To further validate the miR29b targets in diabetic nephropathy, luciferase reporter assay was performed. For this purpose, 3’-UTRs of DNMT3A, DNMT3B and SP1 (as SP1 is the transcription factor of DNMT1) are cloned into downstream region of luciferase reporter gene of pmirGLO vector and named as pmirGLO-DNMT3A, pmirGLO-DNMT3B and pmirGLO-SP1 respectively (S2 Fig). All the ligated clones were transformed into *E coli* DH5α strain for amplification (S2 Fig). All cloned constructs were sequenced by Sanger sequencing (S4 Fig). All the constructs inclusive of miR29b mismatch sequence were co-transfected with miR29b mimics in cells. The obtained results have showd that normalised Luciferase activity of DNMT3A, DNMT3B and SP1 was significantly mitigated in contrast with control group and miR29b mismatch group after transfection with miR29b mimics (Fig 10).

## Discussion

Studies related to DNA methylation have not yet been explored much in kidneys compared with those of other organs affected in diabetes. However, it has been reported that aberrant methylation events occurring at CpG islands in kidney could be associated with progression in DN. Albuminuria is one of the major clinical manifestations of DN. In a recent study, it was found that aberrant methylation patterns could be associated with severity of albuminuria [20]. Moreover, it has been reported that aberrant DNA methylation patterns particularly occurring in proximal tubular cells also contribute to enhance DN conditions [21]. This signifies how aberrant DNA methylation patterns promote development of DN conditions. Diabetic nephropathy also constitutes abundant deposition of ECM proteins which confers to thickening of tubular and glomerular basement membrane. Among several miRNAs, the expression levels of miR29b were found to be downregulated in DN [22]. In addition to this, miR29b has been also claimed to be involved in renal fibrosis and in the regulation of collagen-I, III and IV expressions. However, TGF-β1, a profibrotic cytokine causes downregulation of miR29b via TGF-β1/Smad3 pathway and elevates expression levels of collagen-I, III and IV contributing to renal fibrosis in RPTECs [23].

The relationship between miR29b and DNA methylation were earlier reported in various pathogenic diseases such as in lung cancer [17] and in acute myeloid leukaemia [18]. However, role of miR29b in molecular events underlying DN has not yet been demonstrated. To our knowledge, it is the first evidence suggesting the role of miR29b in regulation of DNMT1, DNMT3A and DNMT3B expressions in RPTECs in DN conditions. DNA hypermethylation is one of the key mechanisms involved in enhancement of DN that is induced by DNMT3A, DNMT3B and transcription factor SP1. In our experiment, through bioinformatics analysis, it was predicted that miR29b potentially targets DNMT3A, DNMT3B and SP1. Moreover, in case of lung cancer and leukaemia this interaction of miR29b with DNMT3A, DNMT3B and SP1 has already been proved. Bioinformatic analysis was carried out using online miRNA target prediction databases including miRDB, TargetScan and FindTar3 which revealed that miR29b could bind to methylation-specific markers (DNMT3A, DNMT3B and SP1). RNA hybrid database have further showed minimum binding energy between 3’-UTR of methylation-specific markers (DNMT3A, DNMT3B and SP1) and mature miR29b, indicating a highly stable complex formation. It has been already reported that the secondary structures of miRNA and mRNA are functionally and thermodynamically essential parameters for the stability of these complexes. From the obtained results, it can be inferred that all targets have minimum free energy below -20kcal/mol and hence, the secondary structure is more stable. These bioinformatics results strongly indicate that miR29b modulates DNA methylation [21].

However, in order to further validate these results, expression profiles of miR29b, DNMT3A, DNMT3B, DNMT1 and SP1 were analyzed in *in vitro* DN model using RPTECs. High glucose is a major risk factor involved in DN progression. It is linked with abnormal activation of protein kinase C (PKC), elevation in expression levels of TGF-β1 and COL4A1 that enhances ECM deposition which causes renal fibrosis in RPTECs [24]. Furthermore, high glucose conditions have induced downregulation of miR29b while overexpression of miR29b triggers cell viability and showed protective action in hyperglycaemic conditions [25]. Ang-II were found to be associated with the induction of proteinuria, renal injury as well as apoptosis and remarkably contributed in the development of chronic kidney disease [26]. Ang-II binds to angiotensin II receptor type 1 (AGTR1) and causes subsequent activation of NF-κB which activates YY1 transcription factor which leads to downregulation of miR29b. [27]. In response to AGE, reduction in miR29b expression level was observed that leads to deposition of collagen in the region of mesangial cells via TGF-β1/Smad3 dependent mechanism [22]. Moreover, it was found that AGE have also induced elevated expressions of NF-κB which can ultimately lead to the reduction in miR29b expression [25]. Moreover, earlier literature suggested that high glucose, Ang-II and AGE product play a vital role in pathogenesis of DN [28, 29]. Thus, in order to mimic DN conditions, we have first developed an *in vitro* DN model mimicing DN microenvironment in RPTECs. Our studies have showed that expression of miR29b was significantly reduced while the expression of DNMT3A, DNMT3B DNMT1 and SP1 (transcription factor allowing transcription of DNMT1) were found to be upregulated in RPTECs based DN model supplemented with HG (30 mM); Ang-II (1 μM) and AGE (150 μg). It had indicated that miR29b downregulation may raise the expression levels of methylation-specific markers (DNMT3A, DNMT3B and DNMT1) and there exists a correlation between miR29b and DNA methylation in DN.

**Fig 11:**
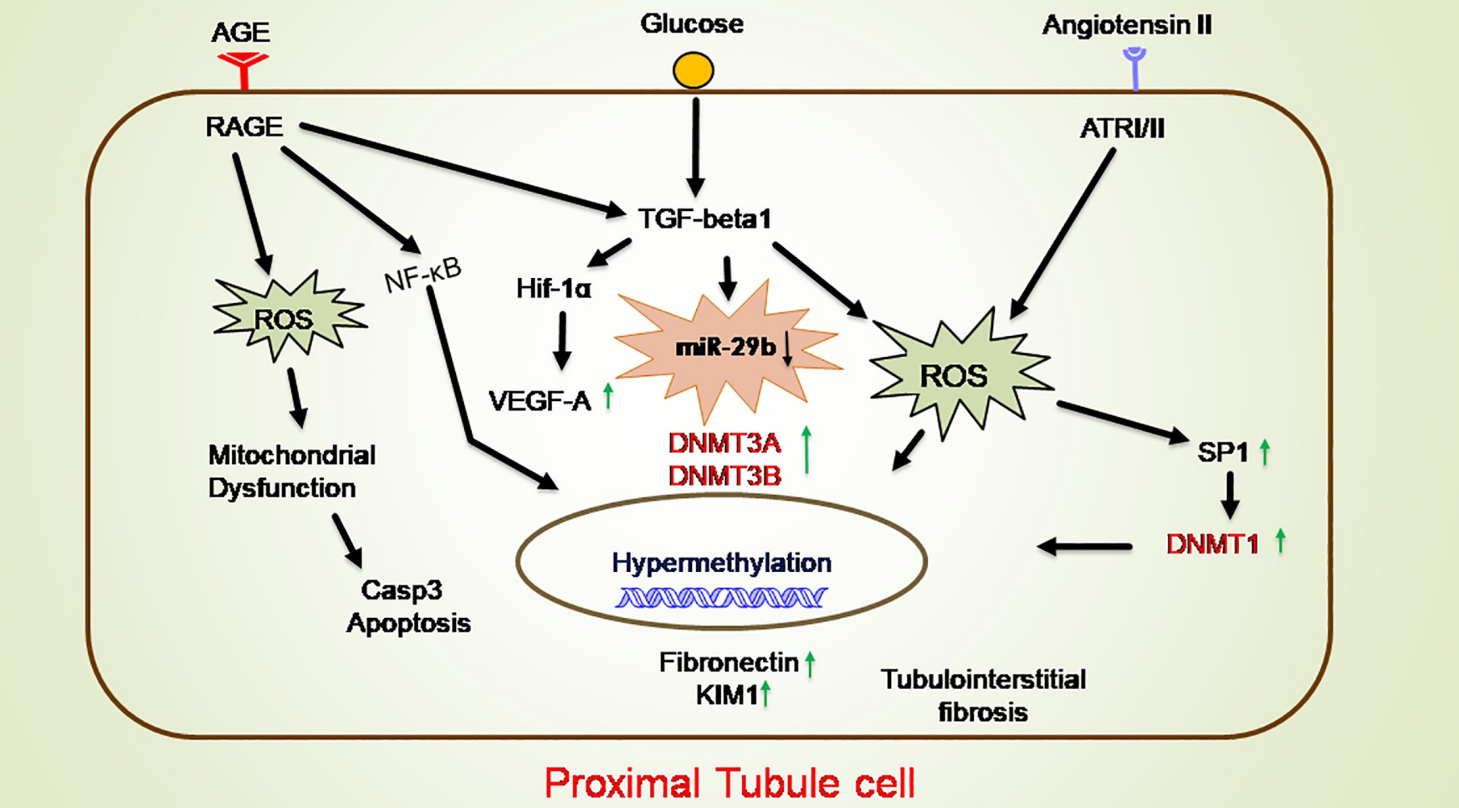
Graphical conclusion. Hemodynamic factor (Ang-II) and metabolic factors (AGE and Glucose) are modulating expression of inflammatory markers like TGF-β1, NF-κB and IL-6 and oxidative stress markers like ROS. Because of increased expression of TGF-β1 there is downregulation of miR29b which leads to dysregulation of methylation and as a result causes hypermethylation of genome.

In our study, when RPTECs were treated with different stress conditions, they were characterized further for the expression of biomarkers involved in DN pathogenesis through qRT-PCR and ELISA. From these results it is also evident that, there is a remarkable rise in mRNA and protein expression levels of TGF-β1, a pro-fibrotic cytokine and VEGF-A, a potent vascular factor participating in inflammation, COL4A1 along with inflammatory markers like IL-6 and NF-κB, apoptotic markers like Caspase 3 and transcription factor Smad2 in RPTECs [30]. Moreover, other pathological conditions were found to be significantly high in developed DN model treated with HG, Ang-II and AGE compared to untreated RPTECs. Expression level of COL4A1 was also elevated which is also found in previously reported studies [23]. Inflammation and oxidative stress conditions seems to play a critical role in enhancement of DN pathogenesis [31]. A gradual increase in ROS was also observed which causes the release of inflammatory cytokines. Superoxide dismutase (SOD) is the leading protector against superoxides in renal tissue during DN conditions. Downregulation of SOD was demonstrated by developed DN model which caused an increase in the cytotoxicity levels of superoxides that contribute to diabetic-induced renal impairment. Therefore, SOD downregulation may be a key factor in development of DN [32]. TGF-β1 have also induced elevation of Smad2 expressions that promoted renal fibrosis via insulin like growth factor binding protein-7 (IGFBP7) [33]. Smad2 mRNA expression profiling was also performed to confirm tubular injury in proximal tubular cells. The study have suggested that Ang-II and AGE products under hyperglycaemic conditions are the key influencers in DN pathogenesis and could provide appropriate DN conditions to study the role of miR29b [34]. These results are consistent with pathogenic conditions of DN and therefore, assessment of miR29b expression levels in this microenvironment could provide a considerable source in establishment of its mechanism in DN (Fig 11) [35].

In this study, transfection of miR29b in developed *in vitro* DN model has showed its active participation in response to hyperglycaemic conditions. Every component of miR29 family possesses distinct physiological function and our hypothesis principally targeted attention towards protective action of miR29b in hyperglycaemia-induced injury of RPTECs in DN. Our experimental results have showed upregulation of miR29b upon transfection with miR29b mimics while its downregulation occurred when miR29b inhibitors were transfected. Further, to validate miR29b potential targets, mRNA expression profiles of DNMT3A, DNMT3B and DNMT1 were analysed which have demonstrated the downregulation of DNA methylation markers upon transfection with miR29b mimics. Transfection of miR29b inhibitors in *in vitro* DN model had demonstrated upregulation in the expression of DNMT3A, DNMT3B and DNMT1. Downregulation of DNMT3A, DNMT3B and DNMT1 and upregulation of the same were also observed at protein level, upon transfection with miR29b mimics and miR29b inhibitors respectively. Besides this, expression levels of biomarkers including KIM-1 and fibronectin were also observed. Among them, KIM-1 is a transmembrane tubular protein that was already reported to be elevated in kidney disease and found to be engaged with inflammation and renal fibrosis in DN [36]. The overexpression of fibronectin was also reported to be the contributor of diabetes-induced renal impairment [37]. In our studies, it was observed that upon transfection with miR29b mimics, alleviation in expression levels of KIM-1 and Fibronectin occurs. From these results, it could be inferred that miR29b could significantly suppress renal injury in proximal tubular epithelial cells in DN conditions. Luciferase reporter assay was performed to further validate whether miR29b could target 3’-UTRs of DNMT3A, DNMT3B and DNMT1 or not. The results obtained from this assay had revealed that miR29b could specifically and successfully target DNMT3A, DNMT3B and DNMT1. This exemplifies the multifarious actions of miR29b in renal injuries.

We can thus conclude that miR29b could effectually target DNMT3A, DNMT3B and DNMT1 contributing in the progression of DN in RPTECs. Therefore, miR29b therapy could be a propitious therapeutic approach towards diabetic nephropathy. The major limitation of this study is the clinical validatation of developed *in vitro* DN model and clinical efficacy of miR-29b in DN. Although miR-29b has been checked for its protective role in diabetic nephropathy, research needs to explore its roles to the fullest.

## Materials and Methods

### Bioinformatic analysis of miRNA29b target prediction

miRNA targets involved in epigenetic modulation of diabetic nephropathy were predicted by using miRNA target prediction tools such as miRDB, TargetScan and FindTar3. These databases were used to identify sequences that are complementary to miRNAs’ seed regions within the human epigenetic regulatory genes. miRDB **(www.mirdb.org)** target prediction program works on high-throughput training datasets and Support Vector Machines (SVMs)[38]. TargetScan **(www.targetscan.org)** finds genes responsible for biological functions that are targeted by miRNAs by searching for the occurrence of conserved 7-mer and 8-mer sites that are complementary to the seed region of that particular miRNA[39]. FindTar3 **(http://bio.sz.tsinghua.edu.cn)** predicts gene sequences targeted by miRNAs on the basis of particular parameters such as free energy of miRNA, target duplex, proper dynamic programming score and seed pairing. The miRNA sequence is recovered from miRBase release 21 (June 2014) while the Refseq gene sequence is recovered from UCSC Genome Bioinformatics. RNAhybrid was used to improve the accuracy of binding site prediction and to determine the secondary structure of miRNA [40].

### Cell culture

Human renal proximal tubular epithelial cells (hRPTECs) were obtained from American Type Culture Collection (ATCC). hRPTECs were maintained in DMEM-F12 (Gibco) with 3.5 μg/mL ascorbic acid (Himedia), 10 ng/mL recombinant human EGF (Invitrogen), 5 pM triiodo-L-thyronine (MPBiomedical), 5.0 μg/mL insulin (Invitrogen), 8.65 ng/mL sodium selenite (Himedia), 5.0 μg/mL human transferrin (Himedia), 25 ng/mL hydrocortisone (Himedia), 1.2 g/L sodium bicarbonate (Sigma Aldrich), 25 ng/mL prostaglandin E1 (Sigma Aldrich), 0.1 mg/mL G418 (Gibco), 100 units/ml penicillin (Gibco), 100 μg/ml streptomycin (Gibco). 25 cm^2^ culture T-flask was used to culture the cells which were stored in a humidified environment at 37ºC in 5% CO_2_ and harvested with trypsin-EDTA (Gibco) when they were in exponential growth phase. Medium was changed every 48 h.

### *In-vitro* diabetic nephropathy model generation

Initially, RPTECs were treated with different concentrations of Ang-II (Sigma Aldrich) (0.1 μM, 0.5 μM and 1 μM) and AGE product (Sigma Aldrich) (50 μg/ml, 100 μg/ml and 150 μg/ml) for 48 h for the generation of in vitro DN model (S1 Table). Three different combinations were tried; HG + Ang-II, HG + AGE and HG + Ang-II + AGE. *In-vitro* DN model was generated using RPTECs to study the role of miR29b in DN pathogenesis. Different stress conditions were generated using glucose, Ang-II and AGE product. Three different combinations (as shown in Table 1) were compared with high glucose model and their characterization studies were performed by RT-PCR to verify the expression profiles of TGF-β1, VEGF-A and COL4A1 involved in DN pathogenesis. In addition to this, qRT-PCR and ELISA were also performed and expression profiles of these biomarkers associated with DN pathogenesis in *in-vitro* DN model were studied in RPTECs.

**Table 1:** Treatment strategy for optimization of *in vitro* DN model.

Treatment Group	AGE (150μg/ml)	Ang II (1μM)	HG (30 mM)	Mannitol(30 mM)
1	-	-	-	-
2	-	-	-	+
3	-	-	+	-
4	-	+	+	-
5	+	-	+	-
6	+	+	+	-

### Transfection of miR-mimics and miR-inhibitors

0.5 x 10^6^ RPTECs were seeded in 6 well plates. After 24 h, miRNA and lipofectamine RNAiMAX (Invitrogen) mixture was prepared as per manufacturer’s protocol. The final concentration of miRNA mimics and inhibitor with respective negative control (scrambled) was 25 pmol per well. miR29b mimics, miR29b inhibitors with their respective negative control were procured from Ambion (Thermo Scientific). After 24 h, media was changed and cells were further incubated for 48 h. The cells were later harvested for further studies. The transfection efficiency was also studied by evaluating miR29b overexpression or knockdown after transfection.

### RNA isolation and real-time PCR

RNA extraction was carried out using Trizol (Invitrogen) reagent and the procedure was followed as given by manufacturer. For mRNA analysis, complementary DNA (cDNA) was synthesized from 1.0 μg of total RNA using iscript cDNA synthesis kit (BioRad) followed by Real-time PCR (StepOne Real time PCR machine Applied Biosystems) which was performed in triplicate with dilution of 1:10 of cDNA using the iQSyber Green supermix (BioRad). KiCqStart Primers (18S, TGF-β1, VEGF-A, COL4A1, NF-κB, CASP3, SMAD2, IL-6, DNMT3A, DNMT3B, DNMT1 and SP1; listed primer sequence in S2 Table) were procured from Sigma Aldrich. Data was taken from the real-time assembly and studied using StepOne software accompanying the PCR machine. All mRNA quantification data was normalized using 18s rRNA as endogenous control. For miRNA analysis, qRT-PCR was performed following TaqMan chemistry as per manufacturer’s directions (AppliedBiosystems). All miRNA data was expressed relative to a U6 small nuclear RNA (U6snRNA). The 2^−ΔΔCt^ method was used to determine the relative expressions of miRNA. All expression studies were carried out in triplicate.

### Western blot

RPTECs lysate from *in vitro* DN model was prepared in RIPA buffer. Estimation of total protein was done using BCA reagent. The proteins were separated in 10% Acrylamide gel and then, transferred over PVDF membrane by using RTA Trans Turbo kit (BioRad). Detection of specific proteins were done by using primary antibody against (DNMT3A 1:2000, Abcam), (DNMT3B 1:250, Sigma), (DNMT1 1:1000, abcam), (KIM-1 1:500, origene), (fibronectin, 1:250 Abcam) and (β-actin 1:5000, Santacruz) at 4˚C overnight. After incubation of primary antibody, membrane was washed with 0.1% TBST and then membrane was incubated with secondary antibody (Goat anti-mouse IgG-HRP 1:20000, Santacruz and Goat anti-rabbit IgG-HRP 1:20000, Abcam) for 1 h. at room temperature. The bands were detected using chemiluminescence substrate (BioRad). Further quantification was carried out by measuring the intensity of each band with the help of ImageJ software (NIH, Bethesda, MD).

### ELISA (Enzyme-linked immunosorbent assay)

Supernatants and cell lysates were collected from *in vitro* DN cell culture model and frozen at -80 ºC until assayed. ELISA of TGF-β1 (RAB0460, Sigma) and VEGF-A (RAB0508, Sigma) was performed as per manufacturer protocol (Sigma). The absorbance was measured at 450 nm in MultiSkanGO UV plate reader (Thermo). TGF-β1 and VEGF-A concentrations were determined from the standard curve normalised with amount of total cell protein. The estimation of COL4A1 was carried out by competitive ELISA Kit as per the manufacturer’s protocol (Bioassay technology laboratory). Standard and sample was incubated with biotinylated antigen in pre-coated plates for 1 h. The plates were thoroughly washed with PBS solution and then incubated with Avidin HRP antibody for 1 h. After complete incubation, wells were again washed with PBS solution and then 50 μl substrate A and substrate B were added. Plates were incubated for 10 mins followed by addition of stop solution. After stopping the reaction, optical density was quantified at 450 nm.

### Immunocytochemistry (ICC)

RPTECs were cultured on the EZ slides (Merck Millipore). Treatment of Ang-II, AGE and HG was given to the cells and subsequent transfection with miR29b mimics and miR29b inhibitors was carried out. The cells were fixed using 4% paraformaldehyde. RPTECs based *in vitro* DN model was incubated with primary DNMT3A antibody (1:1000, Abcam), DNMT3B antibody (4 μg/ml, Sigma), DNMT1 antibody (1 μg/ml, Abcam) for overnight at 4 ºC. After overnight incubation with primary antibody, the slides were washed with PBS (3 times). The slides were then incubated with secondary antibody goat anti-rabbit IgG-FITC for 2 h. After complete incubation, slides were again washed with PBS (3 times) and counter stained with DAPI for cell nuclei. Slides were then observed under the microscope and images were quantified using Image J software.

### ROS detection

The level of ROS (Reactive Oxygen Species) production in *in-vitro* diabetic nephropathy model developed using RPTECs was measured by using the 5-(and 6-)chloromethyl-2′,7′-dichlorodihydrofluorescein diacetate, acetyl ester (H2DCFDA) assay. RPTECs were seeded into a 96-well plate (black bottom) with a density of 1 × 10^5^ cells per well. After incubation for 24 h, the model was generated as per the optimized protocol as discussed above. The cells were then allowed to incubate for upto 48 h and then, 10 μl of 10 mM DCFDA (2’,7’-dichlorofluorescin diacetate) prepared in DMSO was added and the plates were incubated in dark for 1 h. After that cells were washed with PBS (3 times) and finally rinsed with 100μL PBS. The fluorescence of each sample was measured using a Varioskan Lux Multimode Reader (Thermo) with an excitation wavelength of 485 nm and emission wavelength of 535 nm. The samples were measured in triplicate and the data obtained from this analysis was reported in terms of fluorescence intensity in comparison with control sample.

### SOD estimation

The SOD level of cells was determined as per the reported method (Kakkar, Das et al. 1984). RPTECs were seeded with density of 1x10^6^ cells/well in six well plate and incubated for 24 h. After 24 h, the cell homogenization was carried out and centrifuged to collect the supernatant for assay. The mixture for SOD assay consists of 0.1 ml of PMS (PhenazineMethosulphate) (186 μM), 0.3ml of NBT (Nitro Blue Tetrazolium) (300 μM), 1.2ml of sodium- pyrophosphate buffer (0.052 M). The reaction mixture was mixed with 0.2 ml of supernatant and the final volume was made up to 2.8 ml with water. Further, addition of 780 mM NADH was done to begin the reaction and stopped using glacial acetic acid. The intensity of developed chromogen was quantified at 560 nm.

### Luciferase reporter assay

miR29b target sequence and its mismatch sequence from 3’UTR were synthesized (IDT, USA) (S2 Table). Both the annealing oligonucleotides were diluted to 1 μg/μl (Sequence specified in S3 Table). Each oligonucleotide (2 μl) was combined with Oligo annealing buffer (46 μl). It was then heated at 90˚ C for 3 mins and transferred to 37 ˚ C in water bath for 15 mins. The annealed oligonucleotides were used immediately and cloned into the RE sites of XbaI and PmeI pmirGLO plasmid vector (Promega). 50 ng of backbone vector was ligated with 4 ng of insert using Anza T4 DNA Ligase Master Mix. Confirmation of final cloned vector was done via sequencing. HEK-293 cells were transfected with 100 ng pmirGLO-DNMT3A, pmirGLO-DNMT3B and pmirGLO-SP1 vector by using Lipofectamine-2000 reagent (Invitrogen, USA) followed by co-transfection with 10 pmol of miR29b mimics in 24 well plates. Luciferase assay was performed in cell extracts after 48 h of transfection and luciferase activity was quantified using Dual-Luciferase Assay system (Promega, USA). A normalisation of firefly Luciferase activity was carried out by Renilla Luciferase activity.

#### Statistical analysis

Data were expressed as the mean ± Standard deviation. The data for different groups were analysed by one way ANOVA using Dunnett’s multiple test. P value < 0.05 was considered as significant. Statistical analysis was performed using GraphPad Prism Version 5.01.

## Supporting information

S1 Fig Expression profile of TGF-β1, COL-4A1 and VEGF-A by RT-PCR. **A**: mRNA expression profiles of TGF-β1, COL-4A1 and VEGF-A showing a significant elevation at a concentration of 150 μg/ml of AGE in contrast with control group when analyzed through RT-PCR. **B:** mRNA expression profiles of TGF-β1, Collagen type-IV and VEGF-A showing a significant elevation at a concentration of 1 μM of Ang-II in contrast with control group when analyzed through RT-PCR. **C:** mRNA expression profiles of TGF-β1 showing a significant elevation when induced with HG+ Ang-II+AGE in contrast with control group when analyzed through Real-time PCR. **D:** mRNA expression profiles of Collagen type-IV showing a significant elevation when induced with HG + Ang-II + AGE in contrast with control group when analyzed through Real-time PCR. *P<0.05 indicates significant difference vs control; **P<0.01 indicates significant difference vs control; ***P<0.001 indicates significant difference vs control; HG, High glucose; AGE, Advanced glycation end product; Ang-II, Angiotensin-II (n = 3).
(TIF)

S2 Fig **Cloning of Luciferase vector: A.** Clone: E. Coli competent cells + ligated product (vector + respective insert) + Antibiotic, **B.** Positive Control: E. Coli competent cells + No Antibiotic, uniform growth observed, **C.** Negative Control: E. Coli competent cells + Antibiotic, no growth observed.
(TIF)

S3 Fig Confirmation of cloned vector of pmirGLO-DNMTs through Restriction Endonuclease digestion. **A**. Lane description: Lane 1: control pmirGLO-DNMT3A Vector, Lane 2: Reaction control pmirGLO-DNMT3A (without RE), Lane 3: pmirGLO-DNMT3A Vector + Not I, Lane 4: control pmirGLO-DNMT3B Vector, Lane 5: Reaction control pmirGLO-DNMT3B (without RE), Lane 6: pmirGLO-DNMT3B Vector + Not I, Lane 7: 50bp Ladder (Thermo), Lane 8: control pmirGLO-mismatch Vector, Lane 9: Reaction control pmirGLO-mismatch (without RE), Lane 10: pmirGLO-mismatch + Not I **B**. Lane description: Lane 1: λ Hind III Ladder, Lane 2: Clone SP1 digested with Not I, Lane 3: Clone SP1 without Not I, Lane 4: pmirGLO vector digested with Not I, Lane 5: pmirGLO without Not I, 50 bp ladder.
(TIF)

S4 Fig **A: Sequencing analysis of pmirGLO-DNMT3A vector.** Forward and Reverse Oligonucleotides of 3’UTR DNMT3A, **B: Sequencing analysis of pmirGLO-DNMT3B vector.** Forward and Reverse Oligonucleotides of 3’UTR DNMT3B, **C: Sequencing analysis of pmirGLO-DNMT-mismatch vector.** Forward and Reverse Oligonucleotides of pmirGLO-DNMT-mismatch vector, **D: Sequencing analysis ofpmirGLO-SP1 vector.** Forward and Reverse Oligonucleotides of pmirGLO-SP1 vector.
(TIF)

S1 Table Treatment strategy on RPTECs for DN model generation. Three different concentrations of AGE and Ang II were used to optimized the final concentration for treatment.
(DOCX)

S2 Table Primer sequences. Primer sequences of targets
(DOCX)

S3 Table 3’UTR region of miR-29b targets. miR29b targets and their respective 3’UTR regions
(DOCX)
