# Correction: miR29b regulates aberrant methylation in *In-Vitro* diabetic nephropathy model of renal proximal tubular cells

**DOI:** 10.1371/journal.pone.0211591

**Published:** 2019-01-25

**Authors:** Piyush Gondaliya, Aishwarya Dasare, Akshay Srivastava, Kiran Kalia

The images for Figs 6–9 are incorrectly switched. The image that appears as Fig 6 should be Fig 9; the image that appears as Fig 7 should be Fig 6; the image that appears as Fig 8 should be Fig 7; and the image that appears as Fig 9 should be Fig 8. The figure captions appear in the correct order. Please see the correct figures and their respective captions here.

**Fig 6:**
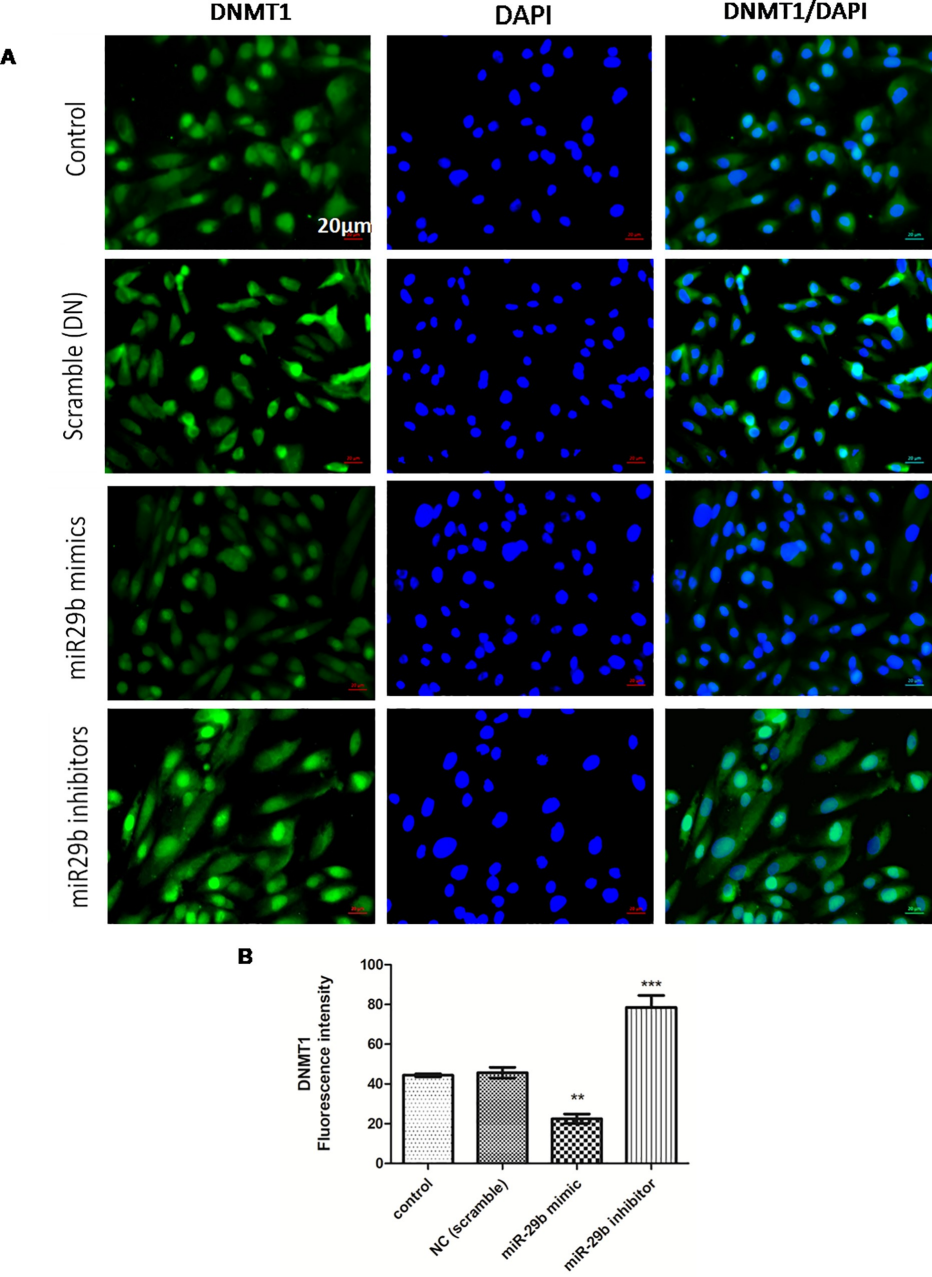
Effect of miR29b mimics and inhibitors on expression profiles of DNMT1 in DN model. **A and B:** Immunofluorescence microscopy as well as quantitative analysis shows that transfection with miR29b mimics attenuated DNMT1 expression level while transfection with miR29b inhibitors showed elevated expression levels of DNMT1. Scale bar, 20 μm. Representative (n = 5), ^*^ indicates ‘p’ value <0.05, ^**^indicates ‘p’ value < 0.01, ^***^ indicate p value <0.001.

**Fig 7:**
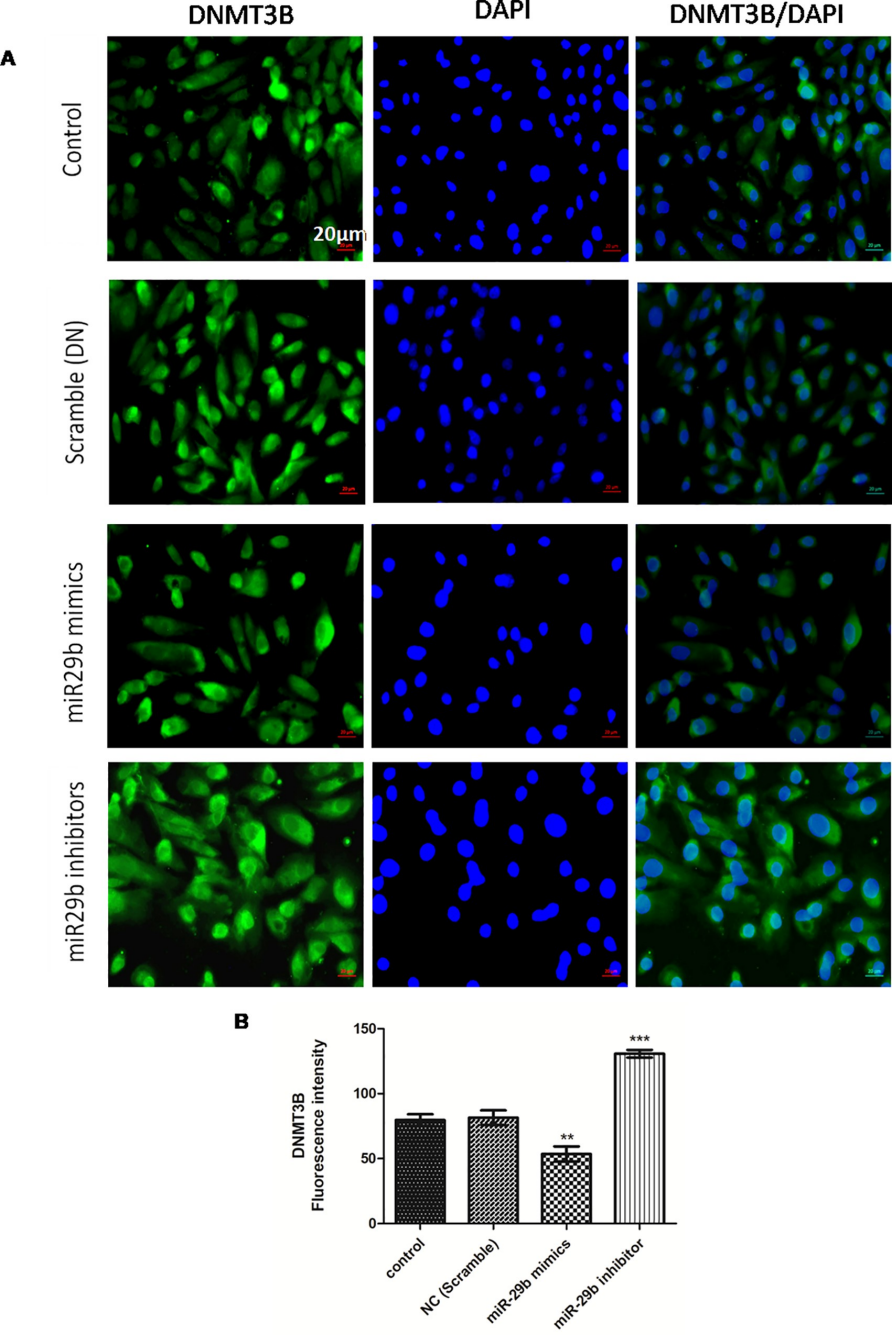
Effect of miR29b mimics and inhibitors on expression profiles of DNMT3B in DN model. **A and B:** Immunofluorescence microscopy as well as quantitative analysis shows that transfection with miR29b mimics attenuated DNMT3B expression level while transfection with miR29b inhibitors showed elevated expression levels of DNMT3B. Scale bar, 20 μm. Representative (n = 5), ^*^ indicates ‘p’ value <0.05, ^**^ indicates ‘p’ value < 0.01, ^***^ indicate p value <0.001.

**Fig 8:**
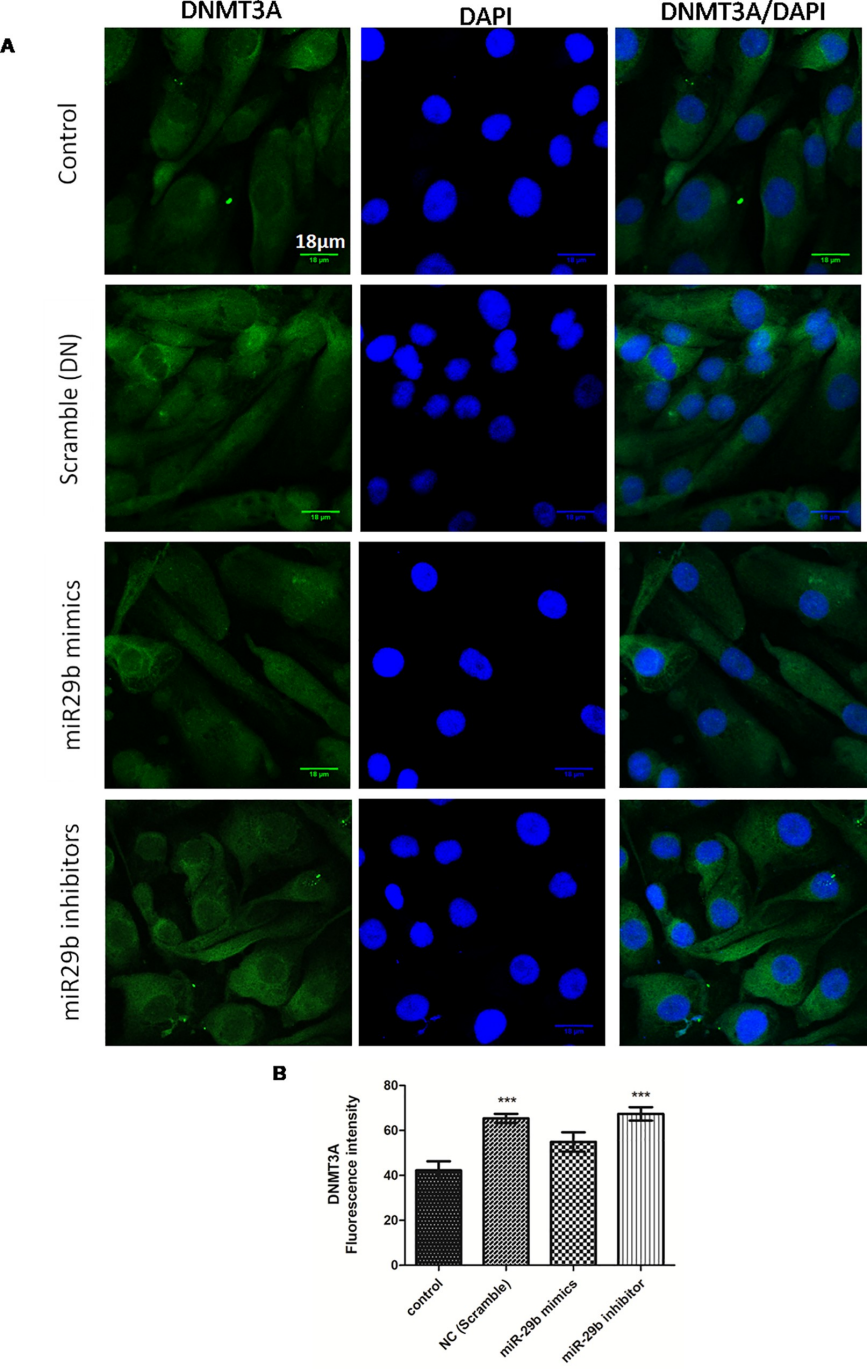
Effect of miR-29b mimics and inhibitor on expression profile of DNMT3A in DN model. **A and B:** Immunofluorescence microscopy as well as quantitative analysis shows that transfection with miR29b mimics attenuated DNMT3A expression level while transfection with miR29b inhibitors showed elevated expression levels of DNMT3A. Scale bar, 18μm. Representative (n = 5), ^*^indicates ‘p’ value <0.05, ^**^ indicates ‘p’ value < 0.01, ^***^ indicate p value <0.001.

**Fig 9:**
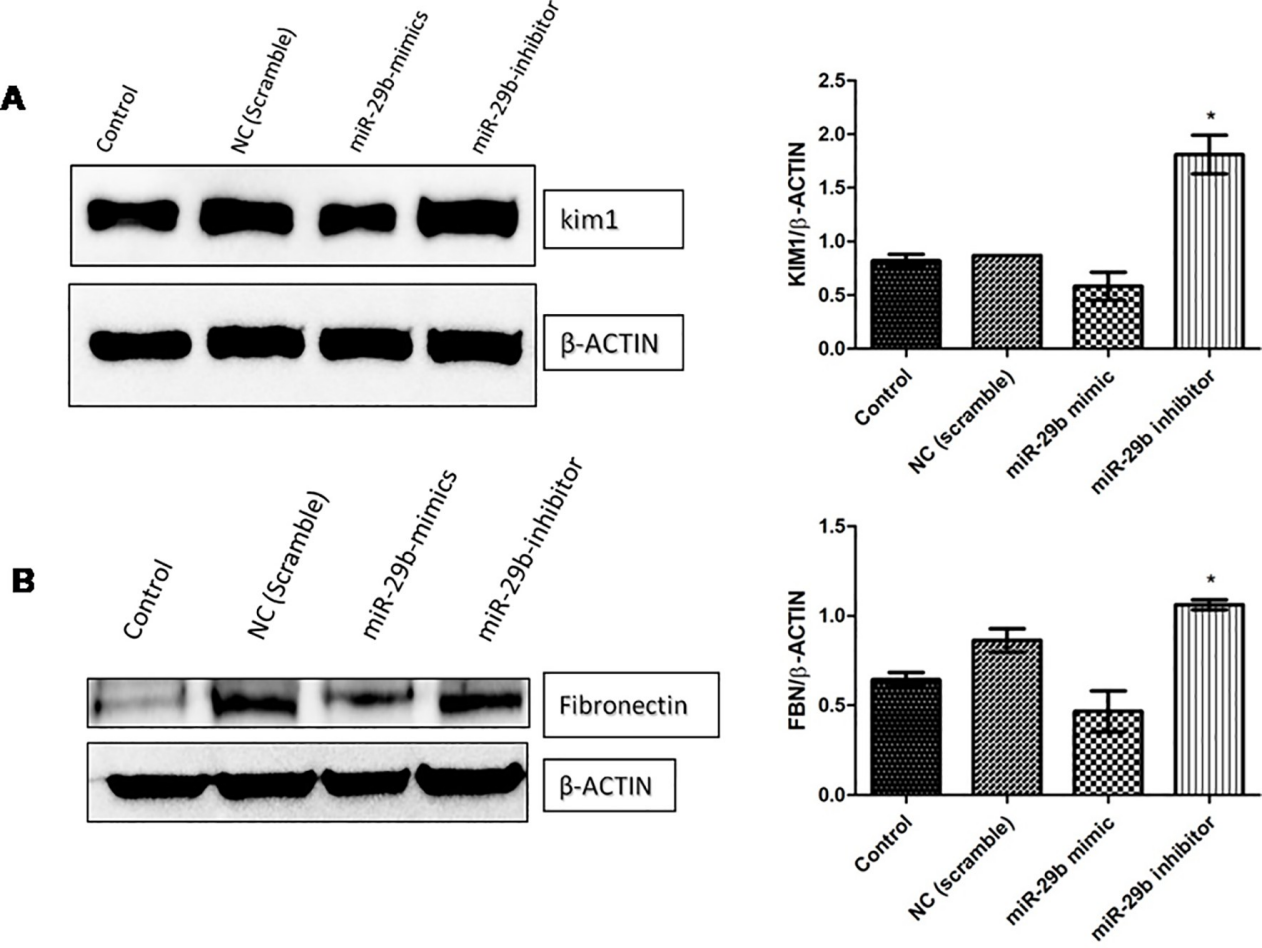
miR-29b decreases fibrosis and tubular injury via downregulation of fibronectin and KIM-1. **A and B:** Effect of miR29b mimics and inhibitors on expression profiles of KIM-1 and Fibronectin in DN model (n = 2), ^*^ indicates p value <0.05.
