# Supplementary figures and images for: miR29b regulates aberrant methylation in In-Vitro diabetic nephropathy model of renal proximal tubular cells
Source: PLoS One. 2018 Nov 29;13(11):e0208044. doi: 10.1371/journal.pone.0208044 (PMC6264835; doi:10.1371/journal.pone.0208044)

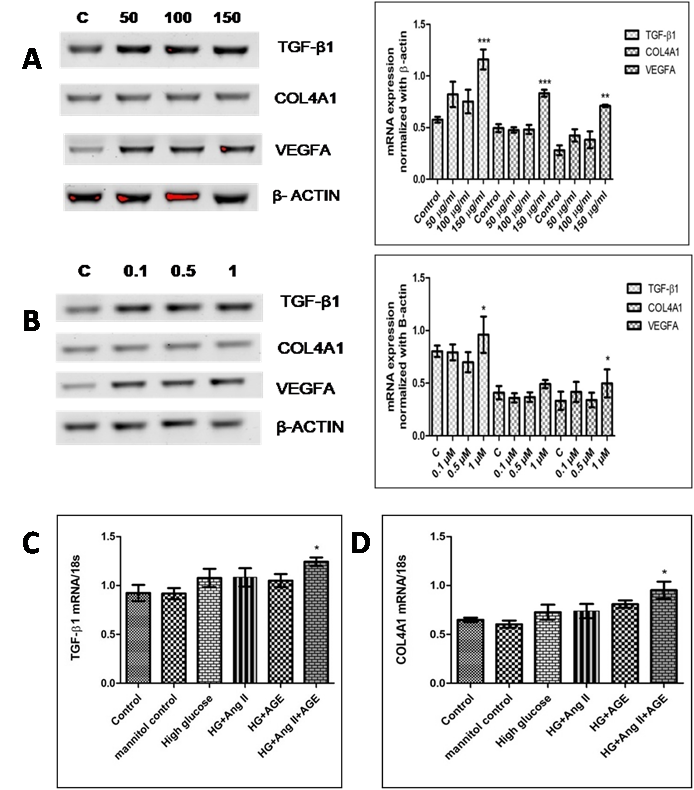

Supplement: S1 Fig — A: mRNA expression profiles of TGF-β1, COL-4A1 and VEGF-A showing a significant elevation at a concentration of 150 μg/ml of AGE in contrast with control group when analyzed through RT-PCR. B: mRNA expression profiles of TGF-β1, Collagen type-IV and VEGF-A showing a significant elevation at a concentration of 1 μM of Ang-II in contrast with control group when analyzed through RT-PCR. C: mRNA expression profiles of TGF-β1 showing a significant elevation when induced with HG+ Ang-II+AGE in contrast with control group when analyzed through Real-time PCR. D: mRNA expression profiles of Collagen type-IV showing a significant elevation when induced with HG + Ang-II + AGE in contrast with control group when analyzed through Real-time PCR. *P<0.05 indicates significant difference vs control; **P<0.01 indicates significant difference vs control; ***P<0.001 indicates significant difference vs control; HG, High glucose; AGE, Advanced glycation end product; Ang-II, Angiotensin-II (n = 3). (TIF) [file pone.0208044.s001.tif]

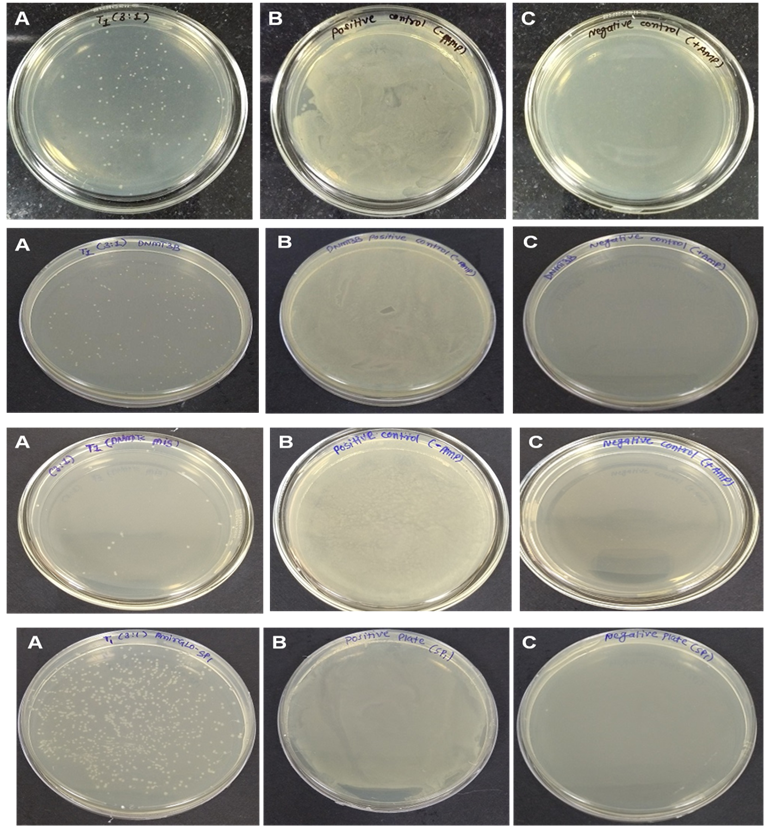

Supplement: S2 Fig — Cloning of Luciferase vector: A. Clone: E. Coli competent cells + ligated product (vector + respective insert) + Antibiotic, B. Positive Control: E. Coli competent cells + No Antibiotic, uniform growth observed, C. Negative Control: E. Coli competent cells + Antibiotic, no growth observed. (TIF) [file pone.0208044.s002.tif]

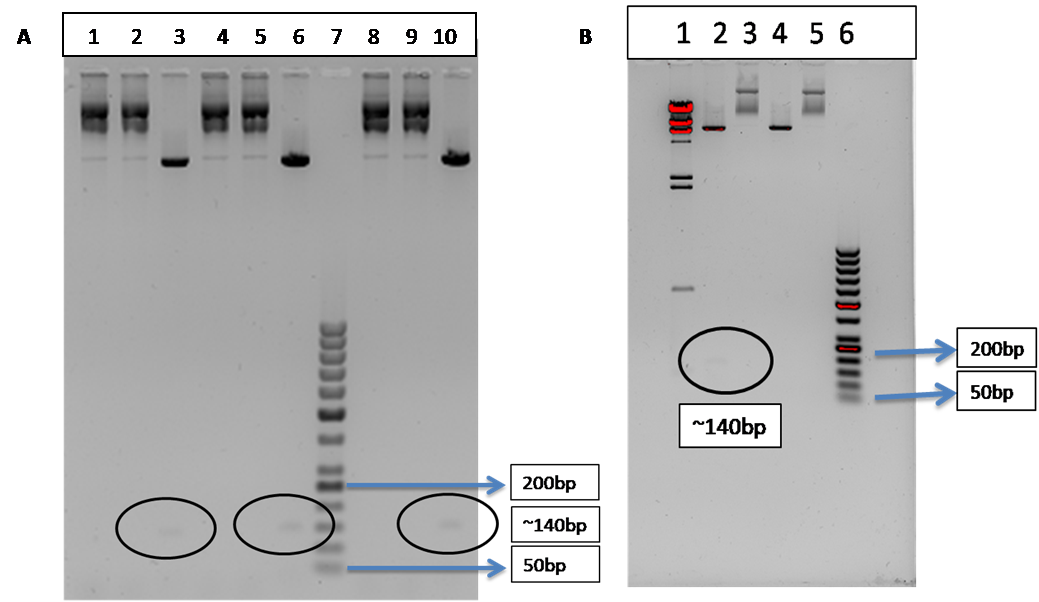

Supplement: S3 Fig — A. Lane description: Lane 1: control pmirGLO-DNMT3A Vector, Lane 2: Reaction control pmirGLO-DNMT3A (without RE), Lane 3: pmirGLO-DNMT3A Vector + Not I, Lane 4: control pmirGLO-DNMT3B Vector, Lane 5: Reaction control pmirGLO-DNMT3B (without RE), Lane 6: pmirGLO-DNMT3B Vector + Not I, Lane 7: 50bp Ladder (Thermo), Lane 8: control pmirGLO-mismatch Vector, Lane 9: Reaction control pmirGLO-mismatch (without RE), Lane 10: pmirGLO-mismatch + Not I B. Lane description: Lane 1: λ Hind III Ladder, Lane 2: Clone SP1 digested with Not I, Lane 3: Clone SP1 without Not I, Lane 4: pmirGLO vector digested with Not I, Lane 5: pmirGLO without Not I, 50 bp ladder. (TIF) [file pone.0208044.s003.tif]

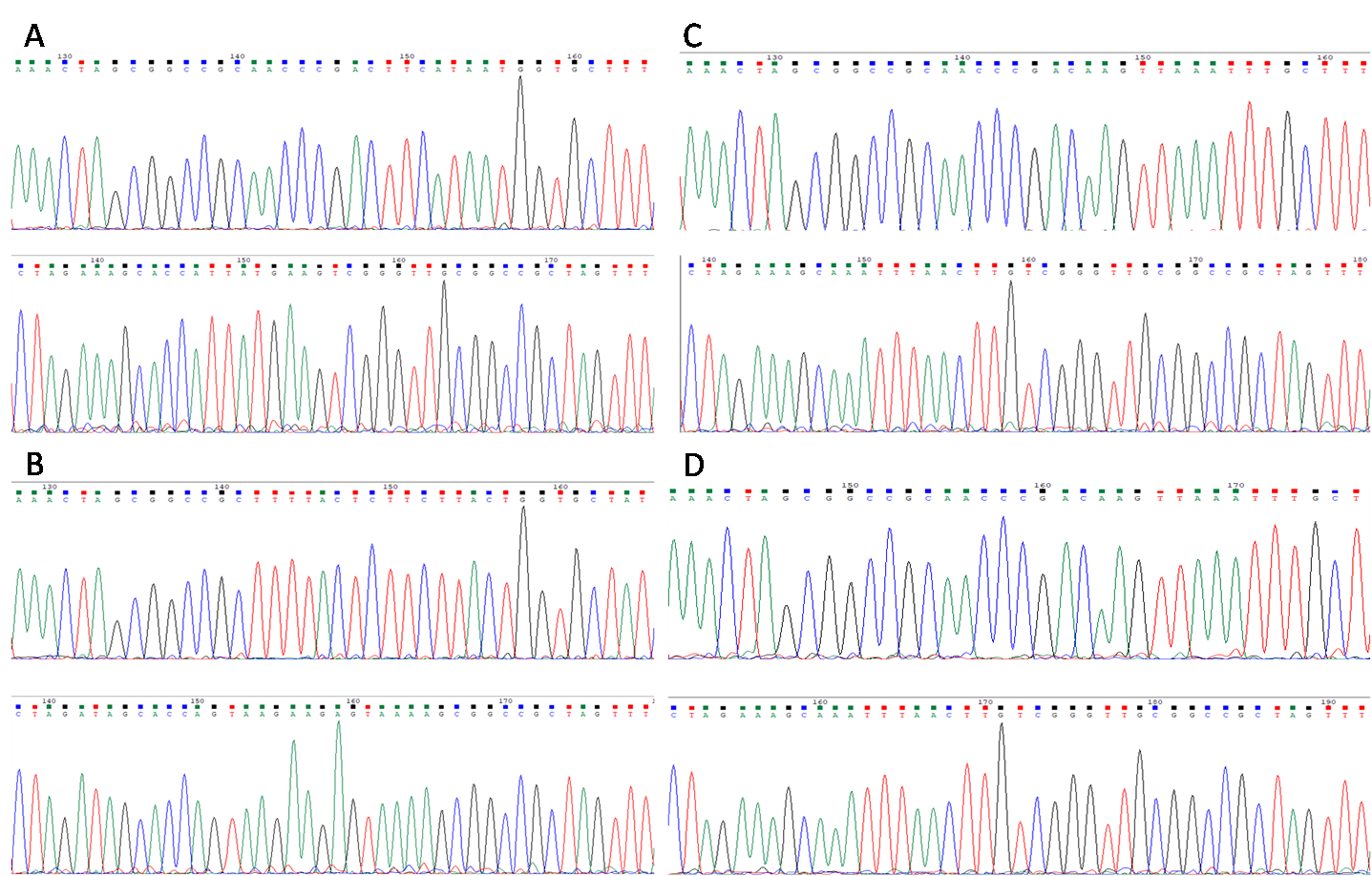

Supplement: S4 Fig — A: Sequencing analysis of pmirGLO-DNMT3A vector. Forward and Reverse Oligonucleotides of 3’UTR DNMT3A, B: Sequencing analysis of pmirGLO-DNMT3B vector. Forward and Reverse Oligonucleotides of 3’UTR DNMT3B, C: Sequencing analysis of pmirGLO-DNMT-mismatch vector. Forward and Reverse Oligonucleotides of pmirGLO-DNMT-mismatch vector, D: Sequencing analysis ofpmirGLO-SP1 vector. Forward and Reverse Oligonucleotides of pmirGLO-SP1 vector. (TIF) [file pone.0208044.s004.tif]
