# Supplementary material for: miR29b regulates aberrant methylation in In-Vitro diabetic nephropathy model of renal proximal tubular cells
Source: PLoS One. 2018 Nov 29;13(11):e0208044. doi: 10.1371/journal.pone.0208044 (PMC6264835; doi:10.1371/journal.pone.0208044)
Supplement: S1 Table — Three different concentrations of AGE and Ang II were used to optimized the final concentration for treatment. (DOCX) [file pone.0208044.s005.docx]

| **Optimization of concentration** | |
| --- | --- |
| **AGE (µg/ml)** | **Ang-II (µM)** |
| **50** | **0.1** |
| **100** | **0.5** |
| **150** | **1** |
| **Final concentration** | |
| **AGE (µg/ml)** | **Ang-II (µM)** |
| **150** | **1** |

**S1 Table: Treatment strategy on RPTECs for DN model generation**
