# Supplementary material for: miR29b regulates aberrant methylation in In-Vitro diabetic nephropathy model of renal proximal tubular cells
Source: PLoS One. 2018 Nov 29;13(11):e0208044. doi: 10.1371/journal.pone.0208044 (PMC6264835; doi:10.1371/journal.pone.0208044)
Supplement: S2 Table — Primer sequences of targets (DOCX) [file pone.0208044.s006.docx]

| **Targets** | **Primer Sequence** |
| --- | --- |
| **18s** | **F:**5’-ATCGGGGATTGCAATTATTC-3’  **R:**5’-CTCACTAAACCATCCAATCG-3’ |
| **Caspase3** | **F:**5’-AAAGCATGGAATGACATC-3’  **R:**5’-CTTTAACACCTTAACTACCG-3 |
| **NF-κβ** | **F:**5’-AAAGCACTGGAATGACATC-3’  **R:**5’-TTTAACACCTTAACTACGC-‘3 |
| **IL-6** | **F:**5’-GCAGAAAAAGGCAAAGAATC-3’  **R:**5’-CTACATTTGCCGAAGAGC-3’ |
| **TGF-β1** | **F:** 5’-AACCCACAACGAAATCTATG-3’  **R:** 5’-CTTTTAACTTGAGCCTCAGC-3’ |
| **VEGF-A** | **F:** 5’-AATGTGAATGCAGACCAAAG-3’  **R:** 5’-GACTTATACCGGGATTTCTTG-3’ |
| **COL4A1** | **F:** 5’-AAAGGGAGATCAAGGGATAG-3’  **R:** 5’-TCACCTTTTTCTCCAGGTAG-3’ |
| **DNMT1** | **F:** 5’-CGTAAAGAAGAATTATCCGAGG-3’  **R:** 5’-GTTTTCTAGACGTCCATTCAC-3’ |
| **DNMT3A** | **F:** 5’-ATTACTACGAGGTCAAACTCC-3’  **R:** 5’-GGGAAACCAAATACCCTTTC-3’ |
| **DNMT3B** | **F:** 5’-CTTACCTTACCATCGACCTC-3’  **R:** 5’-ATCCTGATACTCTGAACTGTC-3’ |
| **SMAD2** | **F:** 5’-AGTGTGTAAAATTCCACCACCAG-3’  **R:** 5’-ATTCTAGTTAGCTGATAGACGG-3’ |
| **SP1** | **F:** 5’-GTGATGGAATACATGATGACAC-3’  **R:** 5’-CCTTCCTTCACTGTCTTTAC-3’ |

**S2 Table: Primer sequences**
