# Supplementary material for: miR29b regulates aberrant methylation in In-Vitro diabetic nephropathy model of renal proximal tubular cells
Source: PLoS One. 2018 Nov 29;13(11):e0208044. doi: 10.1371/journal.pone.0208044 (PMC6264835; doi:10.1371/journal.pone.0208044)
Supplement: S3 Table — miR29b targets and their respective 3’UTR regions (DOCX) [file pone.0208044.s007.docx]

**S3 Table: 3’UTR region of miR-29b targets**

| **Targets** | **3’UTR Oligo** |
| --- | --- |
| **DNMT3A-F** | 5’-AAACTAGCGGCCGCAACCCGACTTCATAATGGTGCTTT-’3 |
| **DNMT3A-R** | 3’-TTTGATCGCCGGCGTTGGGCTGAAGTATTACCACGAAAGATC-‘5 |
| **DNMT3B-F** | 5’-AAACTAGCGGCCGCTTTTACTCTTCTTACTGGTGCTAT-‘3 |
| **DNMT3B-R** | 3’-TTTGATCGCCGGCGAAAATGAGAAGAATGACCACGATAGATC-‘5 |
| **DNMT-MIS-F** | 5’-AAACTAGCGGCCGCAACCCGACAAGTTAAATTTGCTTT’-3 |
| **DNMT-MIS-R** | 3’TTTGATCGCCGGCGTTGGGCTGTTCAATTTAAACGAAAGATC-‘5 |
| **SP1-F** | 5’-AAACTAGCGGCCGCGCTTAGGGGGAGCCCTGGTGCTAT-‘3 |
| **SP1-R** | 3’TTTGATCGCCGGCGTTTGAACTAATTTTGACCACGAAAGATC-’5 |
